# Impact of the *COL1A1* Gene Polymorphisms on Pain Perception in Tennis Elbow Patients: A Two-Year Prospective Cohort Study

**DOI:** 10.3390/ijms252313221

**Published:** 2024-12-09

**Authors:** Paweł Niemiec, Alicja Jarosz, Tomasz Nowak, Anna Balcerzyk-Matić, Tomasz Iwanicki, Joanna Iwanicka, Katarzyna Gawron, Marcin Kalita, Sylwia Górczyńska-Kosiorz, Wojciech Kania, Karol Szyluk

**Affiliations:** 1 Department of Biochemistry and Medical Genetics, Faculty of Health Sciences in Katowice, The Medical University of Silesia in Katowice, Medyków 18, 40-752 Katowice, Poland; alicja.jarosz@sum.edu.pl (A.J.); tnowak@sum.edu.pl (T.N.); abalcerzyk@sum.edu.pl (A.B.-M.); tiwanicki@sum.edu.pl (T.I.); jiwanicka@sum.edu.pl (J.I.); 2 Department of Molecular Biology and Genetics, Faculty of Medical Sciences in Katowice, Medical University of Silesia in Katowice, Medykow 18, 40-752 Katowice, Poland; kgawron@sum.edu.pl; 3 District Hospital of Orthopaedics and Trauma Surgery, Bytomska 62, 41-940 Piekary Śląskie, Poland; marcin.kalita1991@gmail.com (M.K.); karol.szyluk@sum.edu.pl (K.S.); 4 Department of Internal Medicine, Diabetology and Nephrology, School of Medicine with the Division of Dentistry in Zabrze, The Medical University of Silesia in Katowice, 41-800 Zabrze, Poland; skosiorz@sum.edu.pl; 5 Department of Trauma and Orthopedic Surgery, Multidisciplinary Hospital in Jaworzno, Chełmońskiego 28, 43-600 Jaworzno, Poland; wojtekkania@poczta.onet.pl; 6 Department of Physiotherapy, Faculty of Health Sciences in Katowice, Medical University of Silesia in Katowice, Medyków 12, 40-752 Katowice, Poland

**Keywords:** tennis elbow, collagen, *COL1A1*, platelet-rich plasma, tendinopathy, genetic polymorphism

## Abstract

The *COL1A1* gene encodes the α1 chain of type I collagen, and the data reported so far demonstrate that its polymorphic variants may affect biomechanical properties of bones, muscles, and tendons, and contribute to musculoskeletal disorders. Given, however, limited research on these variants in tendon pathology, we analyzed the impact of *COL1A1* polymorphisms on the tendinopathy phenotype and the effectiveness of platelet-rich plasma (PRP) treatment for tennis elbow. Pain perception and therapy outcomes were analyzed from baseline, i.e., before PRP injection to two years post-PRP injection in a cohort of 107 patients. The study focused on seven *COL1A1* variants: rs2249492 (C/T), rs2586488 (A/G), rs2075558 (A/C), rs2253369 (C/T), rs35231764 (A/G), rs1800012 (C/A), and rs9898186 (C/T). We demonstrated that carriers of the TT/CT (rs2249492), AA/AC (rs1800012), and TT/CT (rs9898186) genotypes reported pain related to injury more frequently than subjects with other *COL1A1* variants, also in the context of performing specific activities and other pain characteristics. These polymorphisms did not significantly influence therapy effectiveness, although rs35231764 showed a moderate effect. In conclusion, the T (rs2249492), A (rs1800012), and T (rs9898186) alleles of *COL1A1* gene are risk factors for pain perception in tennis elbow patients, but do not appear to substantially impact PRP treatment outcomes.

## 1. Introduction

Tendinopathies are the most common causes of pain and dysfunction within the musculoskeletal system [1,2,3]. Predisposing factors include prolonged tendon overload, increased physical activity, repetitive movement patterns, and inappropriate work ergonomics. Biological factors which predispose individuals to tendinopathies include age, sex, comorbidities, such as, diabetes, obesity, and hyperlipidemia, and genetic and epigenetic factors [1,2,3].

**Figure 1:**
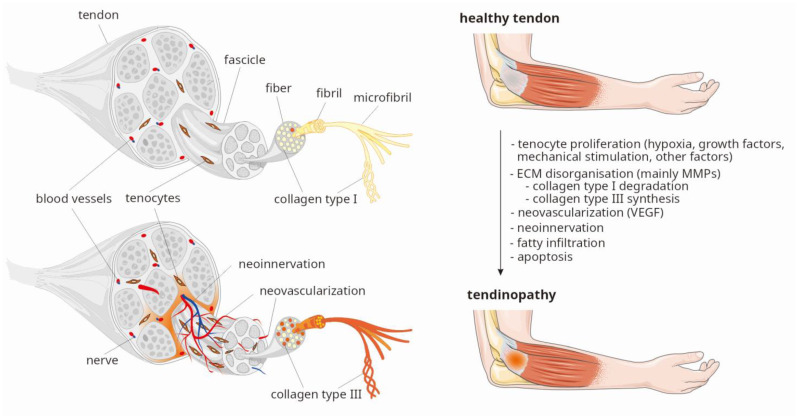
Crucial processes observed in tendinopathy (based on two figures from Servier Medical Art resource [13]: Tendon anatomy and Tendonitis, both licensed under CC BY 4.0., modified by P. Niemiec). Legend: ECM, extracellular matrix; MMPs, matrix metalloproteinases; VEGF, vascular endothelial growth factor.

In response to tendon injury, platelets release growth factors (GFs), such as, vascular endothelial growth factor (VEGF), transforming growth factor (TGF-β), basic fibroblast growth factor (bFGF), platelet-derived growth factor (PDGF), and others [3,4,5,6]. Cytokines, mechanical stimulation, and hypoxia are the primary factors promoting increased tenocyte proliferation, neovascularization and neoinnervation of the damaged tendon areas [1,2,3,4,7,8]. Immediate increase in cellularity is accompanied by thinning and disorganization of the extracellular matrix (ECM), primarily resulting from changes in the ratio of type I collagen (COL1) to type III collagen (COL3), which limits the tendon’s ability to withstand stress [1,9,10,11]. COL1 constitutes a major proteinous component of healthy tendon, making up about 80–95%, while COL3 accounts for approximately 0–5% of its dry mass, respectively. In tendinopathy, an increased degradation of COL1 by collagenases and increased production of COL3 are observed [9,10,11]. It is known that matrix metalloproteinases (MMPs) are responsible for COL1 degradation, and their function is primarily regulated by tissue inhibitors of metalloproteinases (TIMPs) [1,11]. The balance between MMPs and TIMPs is crucial to maintain proper mechanical and biochemical homeostasis of tendons. While GFs release occurs within minutes after injury, inflammation, tenocyte proliferation, ECM disorganization, neovascularization, and neoinnervation occur over a period of several days to several weeks. The entire healing process, associated with tissue remodeling to restore the normal COL1 to COL3 ratio prior to injury can take several months to over a year [11,12]. The main structural changes in the tendon, observed in the first weeks after injury, are shown in Figure 1.

COL1 consists of two α1 polypeptide chains and one α2 chain. The α1 and α2 chains are produced by two separate genes, i.e., the *COL1A1* gene (collagen, type I, alpha-1), located on chromosome 17 (17q21.33) [14], and the *COL1A2* gene (collagen, type II, alpha-1), located on chromosome 12 (12q13.11) [15,16], respectively. Genetic variants of the *COL1A1* are responsible for bone mineral density (BMD) variability and osteoporosis [17], Caffey disease [18], various types of osteogenesis imperfecta [19,20,21,22], and Ehlers—Danlos syndrome [23,24]. Reported data on the role of genetic variation of the *COL1A1* gene in tendon and ligament biology mostly concern one single nucleotide polymorphism (SNP), namely rs1800012 (C/A). SNP rs1800012 is located in the first intron of the *COL1A1*, at the binding site of the transcription factor Sp1. When cytosine is replaced by adenine, the affinity of Sp1 for this site increases, leading to increased expression of the *COL1A1* [25]. Initially, the minor allele A was shown to be associated with decreased risk of tendon injuries [26,27], although the results of reported studies [28,29,30,31,32,33,34], the conclusions of meta-analyses, and critical reviews [35,36] are inconsistent. Other studies demonstrated that in the musculoskeletal system the presence of the A allele appears to have a negative effect on muscle strength [37,38] and is associated with decreased BMD and osteoporosis [25,39,40,41,42], and intervertebral disc degeneration [43].

Tendinopathy at the origin of the extensor carpi radialis brevis muscle (ECRB), commonly known as tennis elbow (TE), accounts for most cases of lateral elbow pain syndrome. One effective treatment method for TE is the injection of autologous platelet-rich plasma (PRP) at the site of injury [44] which, due to its high concentration of growth factors, promotes the healing process. The effectiveness of PRP therapy depends on many factors, including genetic variability, as we previously demonstrated [45,46,47,48,49]. The current work is, therefore, a continuation of a more expanded project, and its primary goal is to evaluate the clinical phenotype and effectiveness of PRP therapy in patients with tennis elbow in the context of the rs1800012 polymorphism and six additional SNPs located in different parts of the *COL1A1* gene, never previously investigated in this area.

## 2. Results

### 2.1. General Characteristics

The research group comprised 107 patients (65 women and 42 men aged 24–64 years, median ± QD: 46.00 ± 5.50) with 132 elbows analyzed, including 25 bilateral cases. The median platelet (PLT) level in the whole blood was equal to 240.00 ± 40.50 (10^9^/l ± QD) and in the PRP reached 343.00 ± 65.00 (10^9^/l ± QD). Females had significantly higher concentration of platelets in whole blood in comparison to males (261.50 ± 33.00 vs. 224.00 ± 38.75, respectively, *p* = 0.000), but PLT concentration in PRP did not differentiate among both sexes (*p* = 0.910). The remaining whole blood and PRP parameters were presented and discussed in our previous works [45,46,47,48,49]. Table 1 summarizes the basic demographic and clinical parameters, including the most common comorbidities, as well as the location of pain radiating from the affected elbow and activities during which it occurred. Elbow pain most often occurred during lifting and radiated to the forearm (Table 1).

### 2.2. Genetic Characteristics

**Figure 2:**
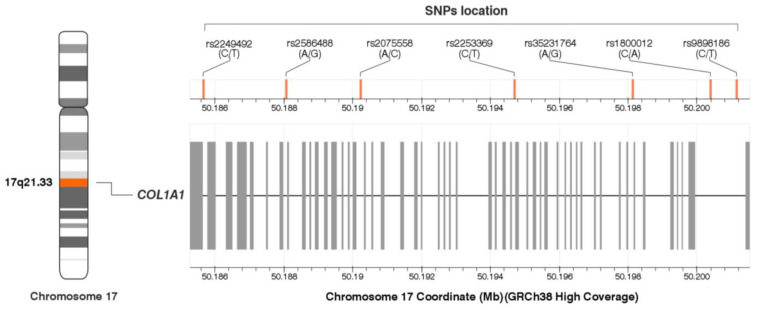
Location of the *COL1A1* gene single nucleotide polymorphisms (SNPs). The figure was created on the basis of data from LDmatrix Tool [50]. Legend: GRCh38, Genome Reference Consortium Human Build 38 Organism: *Homo sapiens*; SNPs, single nucleotide polymorphisms.

The genotype and allele frequencies of the studied *COL1A1* gene polymorphisms are presented in Table 2. Their location in the *COL1A1* gene is shown in Figure 2. The genotype distribution of all polymorphisms was consistent with the Hardy—Weinberg equilibrium (*p* > 0.050).

**Figure 3:**
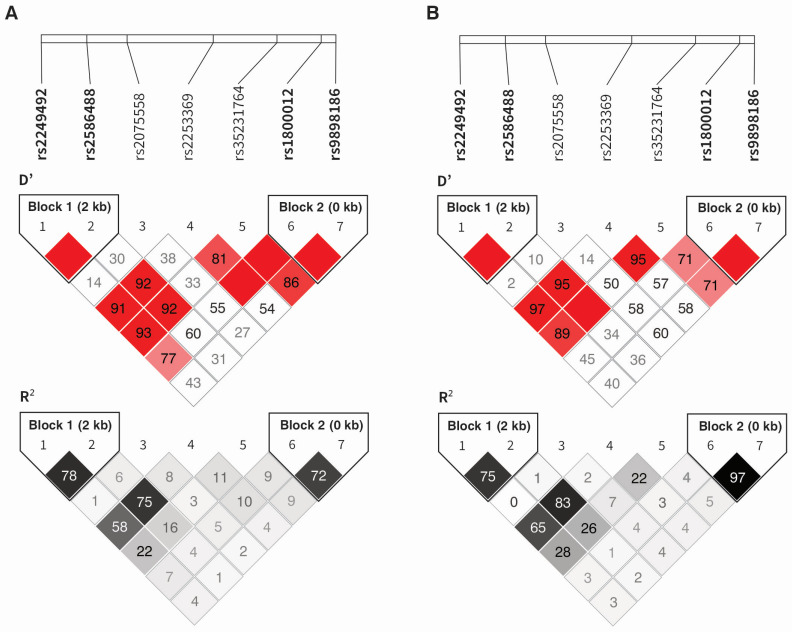
Haplotype analysis of the *COL1A1* gene polymorphisms in the study group (**A**) and CEU population (**B**). The darker the color, the higher the D′ or R^2^ values.

The presence of two haplotype blocks was demonstrated in the study group (Figure 3A). The first one was created by the first two polymorphisms, the second by the last two (in order consistent with their location on the chromosome 17). The remaining polymorphisms were not in linkage disequilibrium, neither with each other nor with the SNPs creating the haplotypes. Similar dependencies characterize the CEU (U.S. Utah residents with ancestry from northern and western Europe, GRCh38) population (Figure 3B).

The TG (rs2249492 and rs2586488, respectively) and CC (rs1800012 and rs9898186, respectively) alleles were in the strongest linkage disequilibrium. The second haplotype was characterized by the highest frequency in the studied group. The existence of a strong association between allelic variants implies that the given alleles are most frequently found in specific arrangements (for example TG and CA for rs2586488 and rs2586488, respectively) (Table 3).

**Figure 4:**
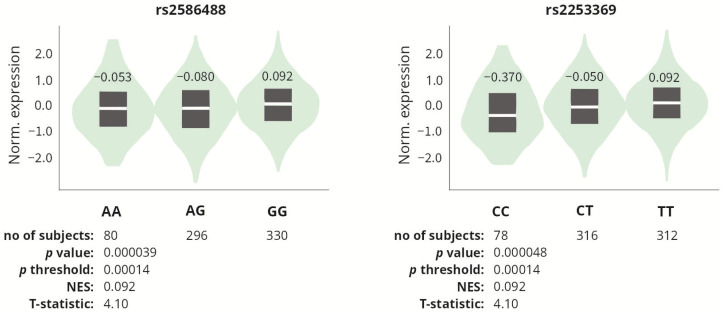
*COL1A1* gene expression in skeletal muscle tissue, depending on the genotypes of the rs2586488 and rs2253369 polymorphisms. Based on the data from GTEx Portal [51].

For each analyzed SNP, in silico analysis of expression quantitative trait loci (eQTL) was performed using the data obtained from the GTEx (The Genotype-Tissue Expression) Portal [51], to determine whether the studied polymorphisms affect *COL1A1* expression. Since the GTEx portal does not contain data on tendons/tenocytes, we analyzed data on skeletal muscle tissue, which anatomically is closely related to the tendon and also derives from mesenchyme. Among the polymorphisms studied, only two affected *COL1A1* gene expression, namely rs2586488 and rs2253369. The GG (rs2586488) and TT (rs2253369) genotypes were associated with increased *COL1A1* expression in the skeletal muscle tissue as compared to its expression in other genotypes (Figure 4).

### 2.3. COL1A1 Gene Polymorphisms and the Effectiveness of PRP Therapy

The effectiveness of PRP therapy was assessed using an additive model and a dominant–recessive model on the basis of comparative analysis of the medians of PROMs (VAS, QDASH, and PRTEE), and their Δ values between *COL1A1* genotype variants, at individual follow-up points after PRP injection. The frequency of patients who achieved the minimal clinically important difference (MCID) for VAS, QDASH, and PRTEE was also compared between variants in two year follow-up period.

In the additive model, four *COL1A1* gene polymorphisms influenced the effectiveness of PRP therapy in univariate analysis (Table 4 and Table S1). It should be noted, however, that in most cases these differences pertained to individual follow-up time points. An exception is rs35231764. Importantly, statistically significant differences were also observed for this SNP before PRP injection (baseline, week 0) and this trend persisted after correction for multiple comparisons. In this case, the absence of differences in delta values for PROMs (ΔVAS, ΔQDASH, and ΔPRTEE) indicates that the rs35231764 polymorphism does not influence therapy effectiveness in the additive model. In relevance to those observations, analyzed *COL1A1* gene polymorphisms are not substantially associated with therapy effectiveness in the additive model.

In the recessive–dominant model, five out of seven analyzed *COL1A1* gene polymorphisms showed differences in response to PRP therapy in the univariate analysis (Table 5). However, the observed differences in PROM values mostly pertained to individual follow-up time points and/or baseline values (week 0), which disqualifies the analyzed polymorphisms as markers of therapy effectiveness (Table 5). Additionally, none of these differences were statistically significant in the multivariate analysis (threshold of significance for the recessive–dominant model: *p* ≤ 0.009). Nevertheless, attention should be drawn to the trend observed for the rs1800012 polymorphism, where worse parameters in terms of pain and functionality were consistently observed in carriers of the A allele. Detailed data for each polymorphism can be found in Tables S2–S8.

**Figure 5:**
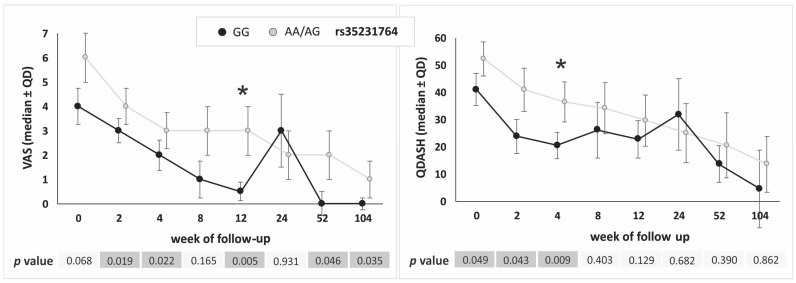
Medians (±QD) of VAS and QDASH values in respect to genotype variants of the *COL1A1* gene rs35231764 polymorphism (recessive–dominant model). Legend: QD, quartile deviation; VAS, visual analog scale; QDASH, quick version of disabilities of the arm, shoulder and hand score; *, differences remaining significant after Hochberg correction for multiple comparisons (threshold of significance for recessive–dominant model: *p* ≤ 0.009).

Similarly to the additive model, in the dominant–recessive model most differences in PROM values were observed between the rs35231764 polymorphism variants (GG vs. AA/AG genotypes), most of the differences being non-significant, however, after correction for multiple comparisons (Figure 5).

**Figure 6:**
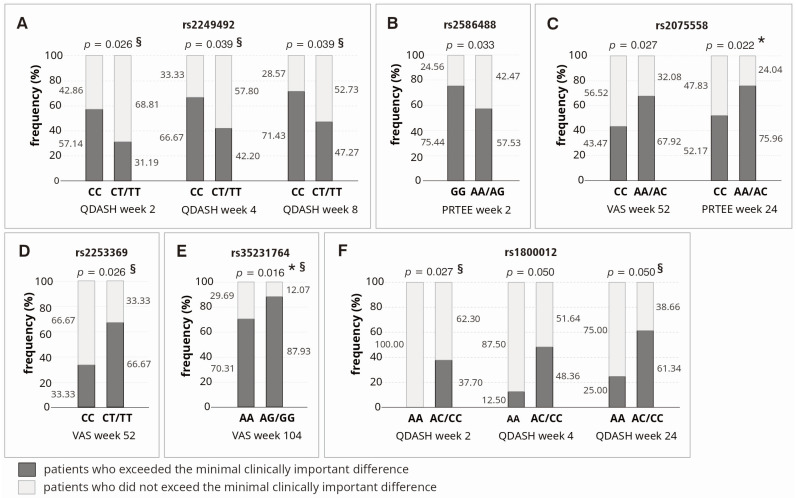
Achievement of minimal clinically important difference for VAS, QDASH and PRTEE in the context of respective polymorphic variants of the *COL1A1* gene: rs2249492 (**A**), rs2586488 (**B**), rs2075558 (**C**), rs2253369 (**D**), rs35231764 (**E**), and rs1800012 (**F**). Legend: PRTEE, Patient-Rated Tennis Elbow Evaluation; QD, Quartile Deviation; QDASH, quick version of Disabilities of the Arm, Shoulder and Hand score; VAS, Visual Analog Scale; *, differences remaining significant after Hochberg correction for multiple comparisons (threshold of significance for MCID analysis: *p* ≤ 0.022); §, differences significant (*p* < 0.050) in multivariate logistic regression analysis (adjusted for all SNPs, age, sex, comorbidities and additional forms of therapy during follow-up).

Studied polymorphisms influenced achievement of minimal clinically important difference (MCID) for analyzed PROMs in dominant–recessive model, in univariate analysis (Figure 6). Homozygotes CC (rs2249492), GG (rs2586488), A allele carriers (rs2075558), T allele carriers (rs2253369), G allele carriers (rs35231764), and C allele carriers (rs1800012) achieved MCID more often than carriers of other genotypes. However, only a few differences were statistically significant after taking into account the correction for multiple comparisons (Figure 6), which confirms the results obtained in the analysis of raw PROMs, suggesting a small effect of *COL1A1* gene variants on the effectiveness of tennis elbow treatment with PRP. In the analysis using another multivariate method, i.e., multivariate logistic regression, more statistically significant differences were identified. However, these differences also concerned individual observation points and selected PROMs (Figure 6).

### 2.4. COL1A1 Gene Polymorphisms and Pain Before Therapy

In the previous section we presented data indicating that statistically significant differences in PROM values between the rs2586488, rs35231764 and rs1800012 polymorphisms variants were also observed before PRP therapy, i.e., at week 0 (Table 4 and Table 5). For this reason, we also investigated whether *COL1A1* gene polymorphisms influence pre-treatment pain perception in other parts of affected limb (radiating pain from the lateral epicondyle of the humerus), also in the context of performing specific activities and other pain characteristics.

The frequency of arm pain differentiated the variants of rs2586488, rs2075558 and rs35231764 only in the univariate analysis (Table 6). The TT homozygotes of the rs9898186 SNP had more often forearm pain than C allele carriers (*p* = 0.016). The frequency of neck pain differentiated variants of the rs2075558 SNP both in additive and recessive–dominant model (*p* = 0.016 and *p* = 0.013, respectively). Regarding pain response to specific motor activities, T allele carriers (rs2249492) were more likely to report pain during lifting than CC homozygotes (*p* = 0.007). The frequency of pain perception, during the day and at night, was significantly higher in carriers of the A allele (rs1800012) than in CC homozygotes (*p* = 0.007 and *p* = 0.000, respectively). Similarly, carriers of the T allele (rs9898186) are more often declared night pain complaints than CC homozygotes (*p* = 0.010). The remaining differences were not statistically significant, after taking into account the correction for multiple comparisons (threshold of significance for pain analysis: *p* ≤ 0.016).

The results of the above analyses, among others indicate, that the risk of pain during lifting was nearly six times higher in carriers of the T allele (rs2249492) compared to CC homozygotes (OR = 5.83, χ^2^ = 9.52). In carriers of the A allele (rs1800012), the risk of pain at night was almost five times higher than in CC homozygotes (OR = 4.86, χ^2^ = 14.26), while the risk of pain during the day was nearly four times higher (OR = 3.87, χ^2^ = 7.20) (Table 6). All these differences showed statistical significance in multivariate analysis after adjusting for age, sex and potential confounding factors, regardless of the multivariate analysis method used.

### 2.5. COL1A1 Gene Polymorphisms and Potential Confounding Factors

The exposure to potential confounding factors (BMI, stimulants, comorbidities, use of additional forms of therapy during follow-up) was compared between individual genotypes of the studied SNPs (Table S9). Only in the case of the rs2075558 polymorphism was it shown that AA homozygotes consumed more units of alcohol per week than C allele carriers (*p* = 0.012). The remaining differences were not statistically significant after taking into account the correction for multiple comparisons.

## 3. Discussion

In the present study, we investigated whether single nucleotide polymorphisms of the *COL1A1* gene influence clinical phenotype and therapeutic effectiveness in patients with tennis elbow treated with platelet-rich plasma. The results of our analysis indicate that patients with the TT/CT (rs2249492), AA/AC (rs1800012), and TT/CT (rs9898186) variants reported pain more frequently at baseline (before the PRP injection), also in the context of performing specific activities and other pain characteristics (pain during the day and/or at night, pain radiating from elbow to other parts of affected limb). This may suggest that these variants are risk factors for pain in patients with tennis elbow, as supported by the calculated odds ratios and relative risk coefficients. Regarding the analysis of PRP therapy effectiveness, we conclude that the studied polymorphisms of the *COL1A1* gene do not substantially influence the efficacy of PRP therapy in tennis elbow patients (statistical significance in single follow-up points, statistical significance at baseline, no trends). However, the rs35231764 polymorphism may be an exception in this context, where a significant trend for VAS was maintained almost throughout the entire observation period (dominant–recessive model, univariate analysis). Below, we discuss the results for each polymorphism individually, or together with the SNP that forms a haplotype with it (in such cases, the identified associations of individual alleles with a specific trait will typically also apply to the haplotype).

**Figure 7:**
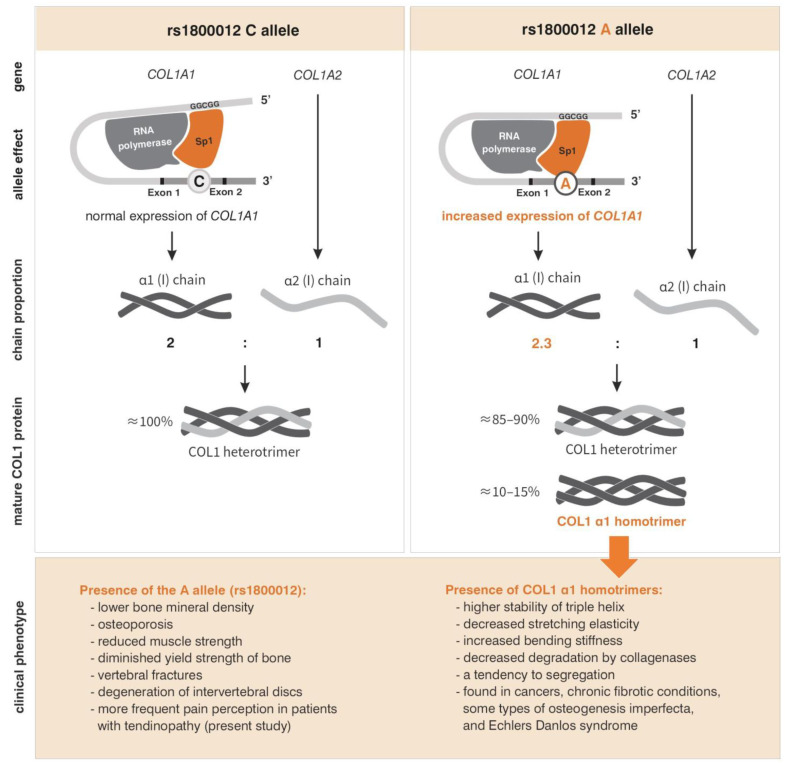
Pathophysiological mechanism for the adverse effects of the A allele (rs1800012) on the musculoskeletal system. Based on [25,37,38,39,40,41,42,43,52,55,56,57,58]. Description in the text.

The polymorphism most extensively studied in the literature is rs1800012. In the musculoskeletal system, an association has been found between the minor allele A and lower bone mineral density, as well as osteoporosis [25,39,40,41,42], reduced muscle strength [37,38], diminished yield strength of bone [52], vertebral fractures [25,52], and degeneration of intervertebral discs [43]. Case-control studies examining the rs1800012 polymorphism [26,27,29,32,53] do not provide an explanation of its role in tendon and ligament damage, which is reflected in the conflicting conclusions of various meta-analyses [35,36,54]. Our results are consistent with functional analyses that identified the A allele as a risk factor for adverse changes in the musculoskeletal system [25,37,38,39,40,41,42,43,52]. These changes may be a consequence of increased *COL1A1* expression observed in A allele carriers [25,52]. The A allele exhibits a greater affinity for binding the transcription factor Sp1 compared to the C allele [25,52]. In CA heterozygotes, there is an increased occurrence of transcripts derived from DNA containing the A allele [25,52], along with a change in the ratio of α1 chain to α2 chain synthesis within the COL1 protein. In CA heterozygotes, this ratio is 2.3:1, compared to 2:1 in CC homozygotes [52], which corresponds to the ratio of both chains in the correct COL1 structure. The proposed pathophysiological mechanism for the adverse effects of the A allele on the musculoskeletal system (Figure 7) suggests that a significant portion of the type I collagen produced by CA heterozygotes and AA homozygotes consists solely of α1 chains [52]. An impaired proportion of α1 and α2 chains in COL1 has previously been observed in cancers, chronic fibrotic conditions, certain types of osteogenesis imperfecta, and Ehlers–Danlos syndrome [55,56,57,58]. Functional studies indicate that COL1 α1 homotrimers exhibit higher overall triple helix stability, decreased stretching elasticity, and increased bending stiffness compared to heterotrimers [58], and are significantly less susceptible to degradation by collagenases [58], meaning that even a small fraction of α1 homotrimers can significantly impact tissue structure. According to this theory, in carriers of the A allele (rs1800012), the accumulation of the COL1 α1 homotrimers, combined with multiple cycles of musculoskeletal tissue remodeling, may exacerbate the changes in bones and tendons, leading to their gradual weakening/degradation.

The impact of the rs1800012 polymorphism on pain perception within the musculoskeletal system remains incompletely understood. It is likely that one of the factors contributing to the higher frequency of pain among carriers of the A allele is the alteration in the biomechanical properties and organization of collagen fibers, resulting from the presence of α1 homotrimers. In vitro studies conducted by Zhang et al. [59] suggest that collagen organization modulates neuronal signaling related to pain in fibrous tissues. The stress on these tissues activates embedded neurons, leading to pain perception. The reduced tensile flexibility and increased bending stiffness of α1 homotrimers [52] appear to be the primary causes. The local kinematics of collagen fibers can be linked to mechano-transduction signaling in neurons, involving extracellular signal-regulated kinase (ERK), which is currently recognized as a critical molecule in pain signaling [60,61]. Our study is not the first to associate the rs1800012 polymorphism with pain perception, but it is the first to do so in the context of tendinopathy. In the study by Navarro-Vera et al., an association of the *COL1A1* rs1800012 SNP and fibromyalgia was identified. Since the observed effect was independent of the patient’s BMD, the authors suggested the involvement of collagen structure in musculoskeletal pain [62]. The rs1800012 polymorphism has also previously been linked to low back pain, where it was implicated in intervertebral disc degeneration [43] and osteoporosis [25,39,40,41,42], which are among the main physical comorbidities of low back pain [63]. These findings suggest that there is no single, straightforward mechanism by which rs1800012 influences pain perception. The effects of its variants on both the biomechanical and physical properties of musculoskeletal tissues can clearly be described as pleiotropic.

Studies suggesting a protective effect of the A allele of the rs1800012 polymorphism in tendon and ligament injuries [26,27,30] were conducted in Caucasian populations. It is worth noting that the minor allele frequency (MAF) varied significantly across individual studies and between patient and control groups, ranging from 13.35% to 23.79% [26,27,29,30,32]. These values differ from the reported frequency of the A allele in European-origin populations (approximately 18.5%) [64]. The selection of reference groups, small sample sizes, and demographic differences may be major factors influencing unintended patient selection, which can affect both allele frequencies and other key results in individual case-control studies. This, in turn, impacts the results of meta-analyses and conclusions of critical reviews [35,36,54,65]. According to Kanyak et al. [35] and John et al. [65], studies included in their critical reviews on the role of genetic variants in anterior cruciate ligament rupture were at high or unclear risk of bias, which was one of the reasons that they were unable to conduct a meta-analysis.

The rs1800012 polymorphism forms a haplotype with rs9898186 (D′ = 100, R^2^ = 72 in the present cohort; D′ = 100, R^2^ = 97 in the CEU population). The CC diplotype (rs1800012 and rs9898186) containing both alleles associated with better prognosis for musculoskeletal disorders was the most common diplotype in our study (0.752), followed by the AT diplotype (0.192). The rs9898186 polymorphism has been studied less frequently, but it has been associated with osteoporosis [66]. In this context, it is not surprising that, in our study, carrying the T allele, as well as TT homozygosity for the rs9898186 polymorphism, were associated with more frequent nocturnal pain and pain radiating from the elbow to the forearm, respectively (with statistically significant differences maintained even after correction for multiple comparisons). The rs9898186 polymorphism did not, however, influence the effectiveness of PRP treatment for tennis elbow.

The next polymorphism analyzed in the present study was rs2249492 (C > T). Our findings indicate that carriers of the T allele experienced more frequent pain during daily activities, such as lifting objects, with statistically significant differences observed after correction for multiple comparisons. This association may result from the fact that T allele carriers exhibit weaker muscle strength than CC homozygotes, as demonstrated in studies involving the knee extensor muscle [38]. Additional research also suggests a link between T allele carriage and malocclusion [67,68]. In this study, the rs2249492 polymorphism, similar to rs2586488 (with which it forms a haplotype), did not impact the effectiveness of TE treatment.

Regarding the other *COL1A1* gene polymorphisms (rs2075558, rs2253369, rs35231764) analyzed herein, there are no other data available in the literature. These polymorphisms moderately influenced pain reported by patients. C allele carriers (AC/CC variants of the rs2075558 SNP) reported pain radiating to the neck more frequently than in the case of AA homozygotes. Additionally, AA homozygotes had significantly higher weekly alcohol consumption, which may explain some of the observed differences. None of these three polymorphisms had a significant effect on the effectiveness of PRP therapy. The rs35231764 polymorphism may be an exception, with GG homozygotes that showed more favorable VAS and QDASH parameters during follow-up (dominant–recessive model) and the observed trend does not seem accidental; however, also in this case, only individual differences between GG and AA/AG variants remained statistically significant after correction for multiple comparisons.

Considering study limitations, first, the sample size is relatively small. Second, the absence of a control group, such as, patients with tennis elbow treated using alternative methods (e.g., physiotherapy), is another limitation. Additionally, the study lacked a standardized post-injection therapeutic protocol. These limitations may have influenced the findings, particularly those related to the effectiveness of the therapy. The small sample size, combined with the low minor allele frequency (MAF) of the rs1800012 polymorphism, resulted in most observed differences between variants displaying statistically insignificant trends after correction for multiple comparisons. Expanding the study group while maintaining the observed distribution of variables would likely render the associations between rs1800012 and therapy effectiveness statistically significant. Since PRP contains numerous cytokines that enhance *COL1A1* expression, including a group of patients not treated with PRP would provide an opportunity to analyze the impact of *COL1A1* polymorphisms on tennis elbow treatment outcomes without the confounding effects of growth factors in PRP, whose pleiotropic effects are challenging to quantify. However, the inclusion of an additional reference group would not alter the main conclusion of this study, i.e., observed association of *COL1A1* polymorphisms with pain perception before treatment. Considering the lack of a standardized post-injection protocol, we deemed it unethical to impose uniform post-therapy regimens on patients due to the varying individual responses to PRP therapy. Confounding factors, such as additional therapies and comorbidities, were monitored during follow-up and accounted for in the statistical analysis of all hypotheses. Given their similar distribution across *COL1A1* gene variants, we believe that these factors did not significantly influence the results. Notable strengths of this work include the ethnically homogeneous study group from the same region (Upper Silesia). Moreover, the follow-up period spanned two years, and the analysis included both under-studied and previously unreported polymorphisms of the *COL1A1* gene, providing insights into gene regions beyond the promoter.

## 4. Materials and Methods

### 4.1. Study Design

This prospective cohort study of tennis elbow patients treated by autologous platelet-rich plasma injection was conducted in accordance with STROBE and MIBO guidelines. Common patient-reported outcome measures (VAS, QDASH, and PRTEE) were collected for two years after single PRP injection (at 2, 4, 8, 12, 24, 52 and 104 weeks). Seven single nucleotide polymorphisms of the *COL1A1* gene were genotyped and the clinical phenotype and efficacy of PRP injection were compared between genotypic variants of SNPs.

### 4.2. Patients

**Figure 8:**
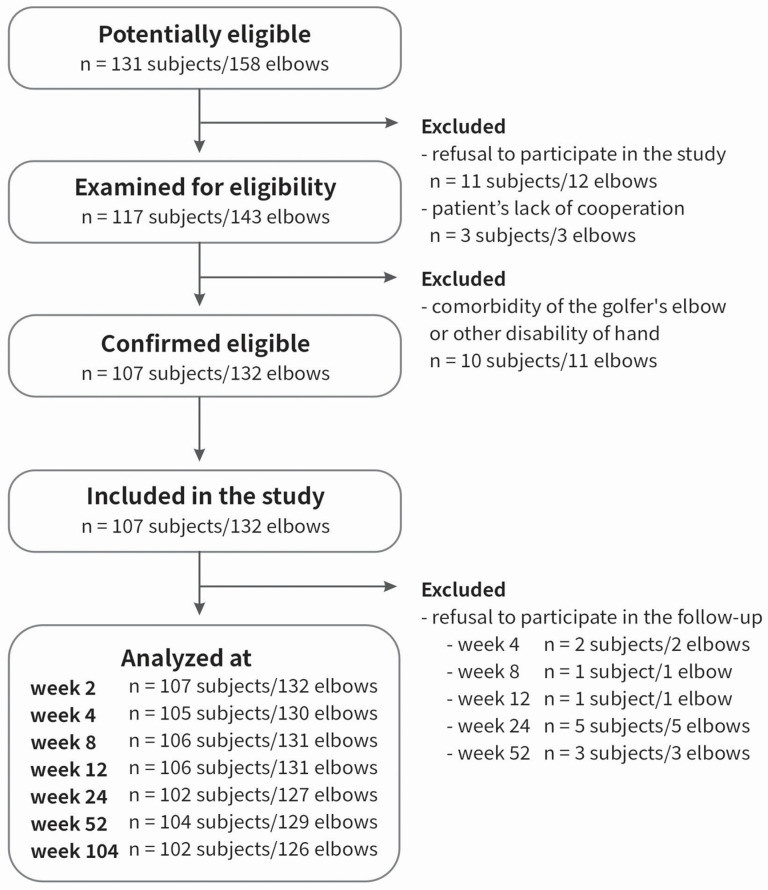
Flowchart of the study selection.

The patient group has been described in detail previously [45,46,47,48,49]. There was a group of 107 patients (132 elbows, 100%), with lateral elbow tendinopathy (M77.1., ICD-10), treated with PRP. The inclusion criteria were as follows: a diagnosis of tennis elbow confirmed by medical history, including pain at the common extensor origin radiating proximally and distally, reduced grip strength, pain and muscle weakness during lifting or holding objects, morning stiffness, positive Thomson’s and Mill’s tests, Cozen’s sign, tenderness over the lateral epicondyle of the humerus, symptoms persisting for at least three months before injection, and treatment with PRP. The exclusion criteria were additional injury/disease of affected limb, prior surgery or PRP injection, steroid injections in the last 6 months, rheumatoid arthritis, pregnancy, active malignancy, cervical radiculopathy, current anti-platelet medication and cognitive limitations. The flow-chart for patient selection is shown in Figure 8.

Patients were enrolled between November 2018 and November 2019 and follow up data were collected until November 2021. There was no formal post-injection rehabilitation protocol. Further post injection therapy (physiotherapy, nonsteroidal anti-inflammatory drugs) was monitored during the follow-up but was not a criterion for exclusion.

### 4.3. PRP Separation and Injection Procedure

All procedures used in this work have been described before [45]. In brief, blood collection, separation and injection of PRP were performed in standardized conditions (20 °C, same light exposure). PRP was separated immediately after blood collection, following the manufacturer’s instructions (Autologous Conditioned Plasma, Arthrex GmbH, München, Germany). The step preceding the separation of PRP was mixing the blood with 3.13% sodium citrate (MediPac GmbH, Königswinter, Germany) in a ratio of 9:1. Immediately after separation, approximately 2.0–3.0 mL of PRP was injected into the common extensor origin area, under ultrasound guidance (Minray DC-3 device, Mindray North America, Mahwah, NJ, USA), using a linear probe (frequency range of 5, 7.5, 10 MHz). After the injection, patients were advised to avoid heavy use of the affected limb for 24 h. No complications, such as infection of the injection site, were observed.

### 4.4. Follow-Up, Outcomes, Measures of Effectiveness

Pre-treatment pain perception (baseline, week 0) in the elbow and other parts of affected limb (radiating pain from the lateral epicondyle of the humerus), also in the context of performing specific activities (lifting, grabbing, bending the wrist, etc.) and other pain characteristics, was examined.

The effectiveness of the therapy was analyzed by comparing clinical condition between patients with individual genotype variants (medians of PROMs: VAS, QDASH, and PRTEE, as well as their Δ values) at 2, 4, 8, 12, 24, 52 and 104 weeks of follow-up, to the condition from the day of PRP injection (week 0). In the case of VAS, the range of pain was defined from 0 (minimum) to 10 (maximum). In the case of QDASH and PRTEE, the range of pain and disability ranged from 0 (minimum) to 100 (maximum).

An additive model (between genotypes) and a dominant–recessive model (between homozygotes of a given allele and carriers of the other allele) were applied. The third method for evaluating therapy effectiveness between genotypes involved comparing the frequency of patients who achieved the minimal clinically important difference (MCID) for VAS, QDASH, and PRTEE at individual follow-up points. The MCIDs for respective PROMs were determined based on literature references. PRP therapy was considered effective when the mean difference in outcome (between the follow-up point and week 0) reached or exceeded 1.5 points for VAS [69], 15.8 points for DASH [70], and 11 points for PRTEE [71]. The frequency of patients who achieved the MCID threshold value and those who did not achieve therapeutic success was calculated separately for each PROM, at each time point of follow-up.

### 4.5. Genetic Analyses

Whole blood for DNA testing was collected on the same day the injection was administered. DNA was isolated from peripheral blood leukocytes using the MasterPure genomic DNA purification kit (Epicenter Technologies, Madison, WI, USA). SNPs of the *COL1A1* gene were genotyped using the TaqMan Predesigned SNP Genotyping Assay kits and the Roche LightCycler^^®^^480II (Roche Diagnostics Corporation, Indianapolis, IN, USA). The accuracy of genotyping was checked by re-genotyping 10–15% of samples. The repeatability of results was 100%.

SNPs with minor allele frequency ≥ 20% in populations of European origin (CEU, U.S. Utah residents with ancestry from northern and western Europe), based on the Database of SNPs of National Center for Biotechnology Information, U.S. National Library of Medicine [64] were selected. There were rs2249492 (C/T), rs2586488 (A/G), rs2075558 (A/C), rs2253369 (C/T), rs35231764 (A/G), rs1800012 (C/A), and rs9898186 (C/T) variants. The frequency of the rs1800012 polymorphism allele is less than 20% in populations of European origin (≈18.5%), but it was included in the study due to its functional nature in the tissues of the musculoskeletal system. Location of SNPs on the *COL1A1* gene is shown in Figure 2. To determine whether the studied polymorphisms affect gene expression, the in silico analysis of expression quantitative trait loci (eQTL) was performed using the data obtained from the GTEx (The Genotype-Tissue Expression) Portal [51].

### 4.6. Statistical Analyses

Data were analyzed using Statistica 13.0 software (TIBCO Software Inc., Palo Alto, CA, USA) and calculator for multiple comparisons [72]. The normality of quantitative data distribution was evaluated using the Shapiro–Wilk test. Given that all examined quantitative variables exhibited a non-normal distribution, results were reported as medians, with quartile deviation (QD) as their spread. Non-parametric tests were employed for comparisons, including the Mann–Whitney U test for dichotomous grouping variables and the Kruskal–Wallis test for grouping variables with more than two categories. Cases with missing data were excluded from the relevant comparisons.

Genetic data were analyzed under additive and dominant–recessive inheritance models. Differences in PROMs and their Δ values were compared between carriers of different genotypes of the studied SNPs. The χ^2^ test was used to assess Hardy–Weinberg equilibrium, as well as to compare genotype variant frequencies across categories of qualitative variables, and to calculate odds ratios and relative risk ratios. Odds ratios (OR) and their 95% confidence intervals (CI) were estimated using a univariate analysis. Risk ratio (RR) values with 95% CI were used when the number of subjects in any of subgroups was 0. Yates’ correction was applied to subgroups with fewer than ten subjects. 

Haplotype blocks in the study group were determined using HaploView 4.2 software (Broad Institute of MIT and Harvard, Cambridge, MA, USA) [73] and the algorithm by Gabriel et al. [74]. Haplotype blocks for the CEU population were defined using the LDmatrix Tool [50]. D′ and R^2^ values were used as measures of linkage disequilibrium. Statistical significance was set at *p* < 0.050, and in instances of multiple comparisons, *p* values were adjusted using the Hochberg correction [67]. The correction was calculated for all SNPs (separately for additive and dominant–recessive models), in relation to specific hypotheses. Each hypothesis (assumed association of SNPs with pain, therapy effectiveness, achievement of MCID) was tested taking into account age and sex, comorbidities (diabetes mellitus, overweight/obesity, hypercholesterolemia, hypertension) and additional forms of therapy during follow-up (physical therapy, manual therapy, NSAIDs). The Hochberg correction was chosen due to the primarily exploratory nature of the analyses conducted, the presence of clearly defined research hypotheses, and the relatively small number of statistically significant variables [67]. An additional method of multivariate analysis used was multiple logistic regression. This approach was applied to binary variables, specifically hypotheses concerning the association of *COL1A1* gene alleles with the clinical phenotype of TE and the achievement of MCID. In this method also, data for all polymorphisms were adjusted for age, sex, comorbidities, and additional forms of therapy administered during an observation period.

## 5. Conclusions

The carriage of the T (rs2249492), A (rs1800012), and T (rs9898186) alleles of the *COL1A1* gene may be considered a risk factor for pain perception in patients with tennis elbow, as supported by findings from previous functional studies. *COL1A1* gene polymorphisms do not appear to substantially impact the effectiveness of PRP treatment for tennis elbow, or their effect is moderate (rs35231764). Regardless, it seems logical to assume that variants influencing pain perception will also affect the course and outcome of therapy. Patients experiencing more severe pain before treatment may not achieve significant improvement following its application. If, in the future, *COL1A1* gene variants (particularly rs1800012) were included in a hypothetical panel of genetic markers for musculoskeletal disorders, it would be reasonable to inform TE patients—regardless of the type of planned therapy—about its potential limitations due to an increased risk of pain perception. Such patients require additional therapeutic options, and it is possible that, in the future, they will have access to more personalized treatment protocols that take into account the mechanical properties of individual components of the musculoskeletal system, including tendons.

This study underscores the influence of *COL1A1* genetic variants on the clinical phenotype of patients with tendinopathies and highlights gaps in our understanding of the role these variants play in the molecular phenotype of tendons determination. Expanding knowledge of the tendon matrisome and transcriptome may, in the future, help improve our understanding of tendon physiology and aid in the selection and optimization of therapeutic strategies.

## Figures and Tables

**Table 1:** Demographic and clinical characteristics of the study group (baseline week 0).

Characteristics			
General	number of subjects, N	107	-
	number of elbows, n (%)	132	(100.00)
	age, median ± QD	46.00	5.50
	BMI, median ± QD	25.65	2.00
	current smokers, n (%)	22	(16.67)
Comorbidities	diabetes mellitus, n (%)	4	(3.03)
	gout, n (%)	8	(6.06)
	obesity (BMI ≥ 30), n (%)	26	(19.70)
	overweight/obesity (BMI ≥ 25), n (%)	86	(65.15)
	hypercholesterolemia, n (%)	10	(7.58)
	hypertension, n (%)	18	(13.64)
Pain of elbow	in LE area, n (%)	132	(100.00)
	during the day, n (%) *	92	(70.80)
	at night, n (%) *	67	(51.54)
	during lifting, n (%) *	117	(90.00)
	when grabbing, n (%) *	80	(61.54)
	when pressing, n (%) *	85	(65.39)
	when bending the elbow, n (%) *	92	(70.77)
	when bending the wrist, n (%) * the wrist	44	(33.85)
Pain radiating to the	wrist, n (%) *	40	(30.77)
	forearm, n (%) *	65	(50.00)
	arm, n (%) *	32	(24.62)
	shoulder, n (%) *	26	(20.00)
	neck, n (%) *	12	(9.23)

Legend: BMI, body mass index; LE, lateral epicondyle of the humerus; QD, Quartile Deviation; *, data available for 130 elbows.

**Table 2:** The frequency of genotypes and alleles of analyzed SNPs of the *COL1A1* gene.

SNP	Chromosome 17 Coordinate (GRCh38)	Genotypes	n (%)	Alleles	n (%)	*p* Value HWE Test
rs2249492	50,185,660	CC	21 (15.91)	C	104 (39.39)	0.983
		CT	62 (46.97)	T	160 (60.61)	
		TT	49 (37.12)			
rs2586488	50,188,065	AA	12 (9.09)	A	87 (32.95)	0.655
		AG	63 (47.73)	G	177 (67.05)	
		GG	57 (43.18)			
rs2075558	50,190,224	AA	39 (29.55)	A	148 (56.06)	0.680
		AC	70 (53.03)	C	116 (43.94)	
		CC	23 (17.42)			
rs2253369	50,194,694	CC	12 (9.09)	C	83 (31.44)	0.914
		CT	59 (44.70)	T	181 (68.56)	
		TT	61 (46.21)			
rs35231764	50,198,128	AA	70 (53.03)	A	190 (71.97)	0.781
		AG	50 (37.88)	G	74 (28.03)	
		GG	12 (9.09)			
rs1800012	50,200,388	AA	8 (6.06)	A	49 (18.56)	0.139
		AC	33 (25.00)	C	215 (81.44)	
		CC	91 (68.94)			
rs9898186	50,201,146	CC	78 (59.09)	C	200 (75.76)	0.568
		CT	44 (33.33)	T	64 (24.24)	
		TT	10 (7.58)			

Legend: GRCh38, Genome Reference Consortium Human Build 38 Organism: *Homo sapiens*; HWE, Hardy–Weinberg equilibrium; SNP, single nucleotide polymorphism.

**Table 3:** Frequency of haplotypes of the *COL1A1* gene in the study group.

SNP				Block Size (kb)	Frequency (%)
rs2249492	rs2586488	rs1800012	rs9898186		
T	G	-	-	2	0.603
C	A	-	-		0.341
C	G	-	-		0.056
-	-	C	C	0	0.752
-	-	A	T		0.192
-	-	C	T		0.056

**Table 4:** Median (±QD) values of PROMs for genotypes of the *COL1A1* gene polymorphisms (additive model).

PROM	Week	Median ± QD			*p* Value			
		rs2249492			Kruskal-Wallis	CC vs. CT	CC vs. TT	CT vs. TT
		CC	CT	TT				
VAS	2	3.00 ± 1.00	5.00 ± 1.50	3.00 ± 1.00	0.012	0.005	0.903	0.018
	4	2.00 ± 1.00	4.00 ± 1.50	3.00 ± 1.00	0.019	0.039	1.000	0.125
			rs2075558		Kruskal-Wallis	AA vs. AC	AA vs. CC	AC vs. CC
		AA	AC	CC				
ΔVAS	104	5.00 ± 2.00	4.00 ± 2.00	2.00 ± 3.00	0.025	0.116	0.037	0.930
		rs35231764			Kruskal-Wallis	AA vs. AG	AA vs. GG	AG vs. GG
		AA	AG	GG				
VAS	0	5.00 ± 2.00	6.00 ± 1.50	4.00 ± 1.50	0.004 *	0.018	0.843	0.026
	2	3.00 ± 1.50	4.00 ± 1.50	3.00 ± 1.00	0.006 *	0.088	0.258	0.010
	4	3.00 ± 1.50	4.00 ± 1.50	2.00 ± 1.25	0.036	0.722	0.169	0.034
	12	2.00 ± 1.50	3.00 ± 2.00	0.50 ± 0.75	0.007 *	0.438	0.059	0.006
QDASH	0	48.86 ± 11.37	59.09 ± 12.50	40.91 ± 11.82	0.006 *	0.038	0.588	0.021
	2	36.36 ± 11.36	50.00 ± 19.32	23.86 ± 12.50	0.017	0.010	0.658	0.011
	4	36.36 ± 10.23	45.45 ± 15.91	20.45 ± 9.66	0.012	0.482	0.084	0.010
PRTEE	0	49.50 ± 10.50	63.00 ± 14.00	38.75 ± 16.25	0.001 *	0.002	1.000	0.032
	2	29.00 ± 12.25	37.00 ± 20.00	17.75 ± 3.88	0.007 *	0.086	0.291	0.012
		rs1800012			Kruskal-Wallis	AA vs. AC	AA vs. CC	AC vs. CC
		AA	AC	CC				
VAS	0	5.00 ± 1.25	7.00 ± 1.50	5.00 ± 1.50	0.027	0.486	1.000	0.025
	12	2.00 ± 1.25	4.00 ± 1.50	2.00 ± 1.50	0.035	0.314	1.000	0.044
PRTEE	0	41.20 ± 7.63	59.00 ± 10.25	50.00 ± 14.00	0.042	0.135	1.000	0.092

Legend: QD, quartile deviation; PROM, patient-reported outcome measure; QDASH, quick version of disabilities of the arm, shoulder and hand score; PRTEE, patient-rated tennis elbow evaluation; VAS, visual analog scale; *, differences remaining significant after Hochberg correction for multiple comparisons (threshold of significance for additive model: *p* ≤ 0.007).

**Table 5:** Median (±QD) values of PROMs for genotypes of the *COL1A1* gene polymorphisms (dominant–recessive model).

SNP	PROM	Week	Median	±QD	Median	±QD	*p* Value
rs2249492			CC		CT/TT		
	VAS	2	3.00	1.00	4.00	1.50	0.017
	ΔQDASH	2	15.91	11.36	4.54	11.37	0.038
rs2586488			AA		AG/GG		
	QDASH	0	60.22	11.47	51.14	13.64	0.039
rs2075558			AA		AC/CC		
	VAS	104	0.00	1.00	1.00	1.50	0.023
	ΔQDASH	104	5.00	2.00	3.75	2.50	0.013
rs1800012			CC		AA/AC		
	VAS	0	5.00	1.50	7.00	1.50	0.023
		12	2.00	1.50	4.00	1.50	0.048
		24	2.00	2.00	3.00	2.25	0.018
	QDASH	12	25.00	17.05	34.09	17.05	0.041
	PRTEE	2	25.00	16.00	38.50	14.00	0.035

Legend: QD, quartile deviation; PROM, patient-reported outcome measure; QDASH, quick version of disabilities of the arm, shoulder and hand score; PRTEE, patient-rated tennis elbow evaluation; VAS, visual analog scale.

**Table 6:** *COL1A1* gene variants and pre-treatment pain perception (data available for 130 elbows).

Pain		SNP	Genotypes, n (%)			*p* Value for Models	
						Additive	Recessive–Dominant
		rs2249492	CC	CT	TT		CT/TT vs. CC
during lifting	Yes		15 (71.43)	58 (95.08)	44 (91.67)	0.007 *	0.007 ^1,^* §
	No		6 (28.57)	3 (4.92)	4 (8.33)		
		rs2586488	AA	AG	GG		AA/AG vs. GG
of arm	Yes		6 (50.00)	17 (27.42)	9 (16.07)	0.036	0.049
	No		6 (50.00)	45 (72.58)	47 (83.93)		
		rs2075558	AA	AC	CC		AC/CC vs. AA
of arm	Yes		5 (13.16)	24 (34.78)	3 (13.04)	0.017	0.008 §
	No		33 (86.84)	45 (65.22)	20 (86.96)		
of neck	Yes		0 (0.00)	11 (15.94)	1 (4.35)	0.016 *	0.013 ^2,^* §
	No		38 (100.00)	58 (84.06)	22 (95.65)		
when grabbing	Yes		18 (47.37)	49 (71.01)	13 (56.52)	0.048	0.033 §
	No		20 (52.63)	20 (28.99)	10 (43.48)		
		rs35231764	AA	AG	GG		AG/GG vs. AA
of arm	Yes		22 (32.35)	9 (18.00)	1 (8.33)	ns	0.032
	No		46 (67.65)	41 (82.00)	11 (91.67)		
		rs1800012	AA	AC	CC		AA/AC vs. CC
at night	Yes		5 (71.43)	25 (78.13)	37 (40.66)	0.000 *	0.000 ^3,^* §
	No		2 (28.57)	7 (21.88)	54 (59.34)		
during the day	Yes		6 (85.71)	28 (87.50)	58 (63.74)	0.026	0.007 ^4,^* §
	No		1 (14.29)	4 (12.50)	33 (36.26)		
		rs9898186	CC	CT	TT		CT/TT vs. CC
at night	Yes		33 (42.31)	27 (62.79)	7 (77.78)	0.026	0.010 ^5,^*
	No		45 (57.69)	16 (37.21)	2 (22.22)		
of shoulder	Yes		9 (11.54)	11 (25.58)	6 (66.67)	ns	0.044
	No		69 (88.46)	32 (74.42)	3 (33.33)		
of forearm	Yes		37 (47.44)	20 (46.51)	8 (88.98)		TT vs. CC/CT
	No		41 (52.56)	23 (53.49)	1 (11.11)	ns	0.016 ^6,^* §
when bending the wrist	Yes		25 (32.05)	13 (30.23)	6 (66.67)		
	No		53 (67.95)	30 (69.77)	3 (33.33)	ns	0.040 §

^1^ OR = 5.83 (95% CI: 1.72–19.70), *p* = 0.002, χ^2^ = 9.52; ^2^ RR = 1.48 (95% CI: 1.30–1.67), *p* = 0.013, χ^2^ = 5.41; ^3^ OR = 4.86 (95% CI: 2.07–11.43), *p* = 0.000, χ^2^ = 14.26; ^4^ OR = 3.87 (95% CI: 1.38–10.85), *p* = 0.007, χ^2^ = 7.20; ^5^ OR = 2.58 (95% CI: 1.24–5.33), *p* = 0.010, χ^2^ = 6.65; ^6^ OR = 8.98 (95% CI: 1.09–74.04), *p* = 0.016, χ^2^ = 5.80; Legend: ns, not statistically significant difference; OR, odds ratio; RR, relative risk; SNP, single nucleotide polymorphism; *, differences remaining significant after Hochberg correction for multiple comparisons (threshold of significance for pain analysis in both additive and recessive–dominant models: *p* ≤ 0.016), §; differences significant (*p* < 0.050) in multivariate logistic regression analysis (adjusted for all SNPs, age, sex, comorbidities and additional forms of therapy during follow-up).

## Data Availability

The original contributions presented in this study are included in the article/Supplementary Material. Further inquiries can be directed to the corresponding author.

## Supplementary Materials

The following supporting information can be downloaded at: https://www.mdpi.com/article/10.3390/ijms252313221/s1.
