# Supplementary material for: Impact of the COL1A1 Gene Polymorphisms on Pain Perception in Tennis Elbow Patients: A Two-Year Prospective Cohort Study
Source: Int J Mol Sci. 2024 Dec 9;25(23):13221. doi: 10.3390/ijms252313221 (PMC11642392; doi:10.3390/ijms252313221)
Supplement: Supplementary file 1 [file ijms-25-13221-s001.zip › ijms-3324126-supplementary.pdf]

Table S1. PROMs values in individuals with particular genotypes of the *COL1A1* gene polymorphisms in additive model.

PROM	week	Genotypes of rs2249492 CC		CT		TT		*P* value			
		Median	±QD	Median	±QD	Median	±QD	Kruskal-Wallis test	CC vs CT	CC vs TT	CT vs TT
VAS	0	5.00	3.00	6.00	1.50	6.00	2.00	0.549			
	2	3.00	1.00	5.00	1.50	3.00	1.00	0.012 *	0.005	0.903	0.018
	4	2.00	1.00	4.00	1.50	3.00	1.00	0.019 *	0.039	1.000	0.125
	8	3.00	1.50	4.00	2.00	3.00	1.50	0.293			
	12	3.00	1.50	3.00	2.00	2.00	1.50	0.392			
	24	1.00	2.00	3.00	2.00	2.00	2.00	0.394			
	52	3.00	2.00	2.50	2.50	1.00	1.50	0.419			
	104	1.00	1.50	1.00	1.50	1.00	1.00	0.935			
ΔVAS (vs week 0)	2	1.00	2.00	1.00	1.00	2.00	1.50	0.137			
	4	2.50	2.00	2.00	1.50	2.00	1.50	0.509			
	8	2.00	2.00	2.00	2.00	3.00	1.50	0.873			
	12	2.00	1.50	3.00	2.00	3.00	1.50	0.772			
	24	2.00	2.00	2.50	2.00	3.00	2.00	0.713			
	52	2.00	2.50	4.00	2.50	4.00	2.00	0.587			
	104	2.50	2.25	4.50	2.50	4.00	2.00	0.624			
QDASH	0	55.00	10.23	50.00	15.91	52.27	12.64	0.747			
	2	31.82	11.36	47.73	18.18	40.91	13.64	0.082			
	4	31.82	7.95	38.64	15.91	36.36	13.64	0.320			
	8	25.00	14.77	29.55	15.91	36.36	21.59	0.463			
	12	18.18	17.05	27.27	17.05	31.82	17.05	0.575			
	24	11.36	24.43	25.00	20.45	27.27	20.45	0.884			
	52	13.64	25.00	21.59	21.59	13.64	22.73	0.840			
	104	13.64	21.59	9.09	21.02	15.91	17.05	0.419			
ΔQDASH (vs week 0)	2	15.91	11.36	2.27	11.86	6.81	12.84	0.060			
	4	20.45	11.37	10.22	18.24	9.09	14.77	0.193			
	8	31.81	14.77	13.63	17.05	11.36	18.18	0.150			
	12	27.27	15.89	18.18	15.91	13.64	18.18	0.281			
	24	38.61	16.48	17.04	18.18	18.18	26.14	0.360			
	52	34.09	20.23	22.73	19.32	20.45	21.59	0.640			

	104	40.90	19.32	29.54	23.87	31.82	17.04	0.528			
PRTEE	0	42.50	10.00	53.25	11.25	52.50	14.50	0.542			
	2	27.00	12.50	37.50	18.00	24.50	12.50	0.040	0.133	1.000	0.096
	4	23.00	7.25	29.50	18.00	23.00	12.00	0.106			
	8	19.50	11.50	24.00	18.00	22.00	17.00	0.532			
	12	13.50	15.00	20.50	15.75	20.00	14.75	0.804			
	24	8.00	20.25	17.00	14.75	13.50	17.25	0.688			
	52	6.00	15.25	14.50	15.50	11.50	13.75	0.623			
	104	10.00	14.00	7.00	14.38	7.50	11.50	0.804			
ΔPRTEE (vs week 0)	2	17.50	10.00	12.75	13.00	19.50	11.25	0.067			
	4	22.00	11.50	18.00	12.75	25.00	14.25	0.506			
	8	29.50	12.00	25.50	16.50	25.50	14.45	0.978			
	12	24.00	18.25	29.50	14.50	27.00	15.50	0.679			
	24	35.00	17.63	31.00	18.63	27.40	19.50	0.919			
	52	28.50	15.50	35.50	19.25	31.00	14.25	0.738			
	104	37.00	14.75	38.00	16.00	39.00	15.75	0.443			

**PROM**	**week**	**Genotypes of rs2586488**						***P* value**			
		**AA**		**AG**		**GG**					
		**Median**	**±QD**	**Median**	**±QD**	**Median**	**±QD**	**Kruskal-Wallis test**	**AA vs AG**	**AA vs GG**	**AG vs GG**
VAS	0	7.00	2.13	6.00	1.50	6.00	2.00	0.791			
	2	3.00	1.50	4.00	2.00	4.00	1.50	0.405			
	4	3.00	1.50	3.00	1.50	3.00	1.00	0.472			
	8	3.00	1.00	3.00	2.00	3.00	1.50	0.694			
	12	3.50	1.25	2.00	2.00	2.00	1.50	0.321			
	24	2.00	2.00	2.00	2.00	2.00	1.50	0.580			
	52	3.00	2.75	1.00	2.00	1.00	2.00	0.799			
	104	2.00	1.50	0.00	1.50	1.00	1.00	0.693			
ΔVAS (vs week 0)	2	1.25	1.75	1.00	1.50	2.00	1.50	0.499			
	4	2.75	2.00	2.00	2.00	2.00	1.50	0.637			
	8	1.75	2.50	2.00	2.00	3.00	1.50	0.836			
	12	2.00	2.50	2.50	2.00	3.00	2.00	0.873			
	24	3.00	3.00	2.00	1.50	3.00	1.50	0.517			
	52	1.00	3.75	4.00	2.50	3.00	2.00	0.785			
	104	4.00	3.50	4.00	2.50	4.00	2.00	0.870			
QDASH	0	60.22	11.47	47.72	13.64	52.27	13.64	0.113			
	2	36.36	10.23	39.77	15.91	40.91	15.91	0.967			

	4	38.64	9.09	32.95	17.05	36.36	14.77	0.886			
	8	35.23	17.05	29.55	19.32	36.36	18.18	0.723			
	12	31.82	22.16	27.27	15.91	31.82	17.05	0.427			
	24	31.82	29.55	23.86	22.73	27.27	20.45	0.713			
	52	13.64	27.84	15.91	23.86	22.73	22.73	0.642			
	104	25.00	21.59	4.55	17.05	15.91	20.45	0.124			
ΔQDASH (vs week 0)	2	17.04	19.32	4.54	12.59	6.81	11.84	0.298			
	4	19.31	15.34	11.36	18.30	11.36	12.50	0.418			
	8	30.68	17.61	13.63	18.18	15.91	18.18	0.349			
	12	30.68	17.49	22.72	15.91	15.90	17.05	0.598			
	24	38.58	19.32	20.45	19.32	18.18	21.46	0.598			
	52	38.64	20.34	27.27	21.59	18.17	21.59	0.323			
	104	47.73	20.34	29.55	22.73	29.54	17.04	0.218			
PRTEE	0	49.75	17.50	52.50	11.25	52.50	14.50	0.958			
	2	31.50	6.63	34.25	19.00	27.50	11.00	0.543			
	4	26.50	9.50	24.50	15.75	24.50	11.75	0.801			
	8	25.25	15.63	21.50	19.25	24.00	13.75	0.631			
	12	26.25	17.75	16.00	16.50	21.00	14.00	0.555			
	24	19.50	36.75	15.50	17.50	13.50	17.25	0.704			
	52	8.50	17.00	11.00	16.75	13.00	15.25	0.922			
	104	14.75	15.13	3.00	12.50	8.00	12.25	0.330			
ΔPRTEE (vs week 0)	2	13.25	8.63	14.00	13.50	17.50	9.75	0.277			
	4	18.50	15.13	20.50	13.00	22.00	11.50	0.858			
	8	15.00	20.88	26.75	16.50	27.50	14.25	0.666			
	12	10.00	22.63	29.50	14.50	27.00	14.00	0.426			
	24	19.00	23.25	31.50	17.00	30.50	18.50	0.820			
	52	31.75	24.25	37.75	19.00	29.00	15.25	0.713			
	104	33.25	25.13	38.00	15.50	38.50	15.75	0.840			

**PROM**	**week**	**Genotypes of rs2075558**						***P* value**			
		**AA**		**AC**		**CC**					
		**Median**	**±QD**	**Median**	**±QD**	**Median**	**±QD**	**Kruskal-Wallis test**	**AA vs AC**	**AA vs CC**	**AC vs CC**
VAS	0	6.00	2.00	6.00	1.50	4.00	2.00	0.103			
	2	3.00	2.00	4.00	1.50	3.00	1.00	0.530			
	4	3.00	1.50	3.00	1.50	3.00	1.50	0.514			
	8	4.00	2.00	3.00	1.50	2.00	2.00	0.608			
	12	2.00	2.50	3.00	2.00	2.00	1.50	0.404			

	24	2.00	2.00	2.00	2.00	2.00	1.50	0.842			
	52	1.00	2.00	2.00	2.00	2.00	2.50	0.395			
	104	0.00	1.00	1.00	1.50	1.00	1.50	0.058			
ΔVAS	2	2.00	1.50	1.00	1.50	1.00	2.00	0.384			
(vs week 0)	4	2.00	1.50	2.00	2.00	2.00	2.00	0.567			
	8	3.00	2.00	3.00	2.00	2.00	1.50	0.415			
	12	4.00	2.00	2.00	2.50	3.00	1.50	0.708			
	24	3.00	1.50	3.00	2.50	2.00	1.50	0.411			
	52	4.50	2.00	3.00	2.75	1.00	2.00	0.070			
	104	5.00	2.00	4.00	2.00	2.00	3.00	0.025 *	0.116	0.037	0.930
QDASH	0	45.45	12.50	57.95	13.64	52.27	14.77	0.030	0.079	1.000	0.127
	2	40.91	17.05	43.18	17.05	36.36	13.64	0.226			
	4	31.82	15.91	38.64	13.64	36.36	14.77	0.204			
	8	34.09	22.73	34.09	14.77	25.00	19.32	0.397			
	12	22.73	23.86	32.95	14.77	27.27	18.18	0.182			
	24	15.91	15.91	29.55	21.59	22.73	19.32	0.426			
	52	11.36	23.86	20.45	21.59	25.00	22.73	0.353			
	104	5.68	11.36	20.45	21.59	6.82	21.59	0.157			
ΔQDASH	2	4.54	11.37	6.81	16.48	9.09	11.36	0.880			
(vs week 0)	4	6.82	13.64	16.59	16.48	11.36	12.50	0.432			
	8	11.36	15.91	18.18	17.05	4.54	27.27	0.459			
	12	18.17	18.18	18.18	12.61	4.54	23.86	0.668			
	24	20.45	13.64	25.00	22.73	15.63	22.73	0.634			
	52	22.72	14.77	25.00	19.39	18.17	25.00	0.508			
	104	30.69	20.45	30.68	20.96	27.27	25.00	0.779			
PRTEE	0	49.50	12.00	53.75	15.25	46.00	11.50	0.099			
	2	33.50	15.75	29.00	16.75	27.00	12.75	0.366			
	4	24.50	17.00	26.50	13.50	24.00	8.25	0.663			
	8	24.00	16.75	24.25	15.25	15.50	17.00	0.358			
	12	15.00	20.75	22.75	14.25	16.00	13.00	0.289			
	24	11.00	17.00	19.25	17.00	16.00	15.50	0.473			
	52	5.50	18.50	13.00	14.25	15.00	9.75	0.510			
	104	1.75	7.50	10.00	13.50	5.00	16.25	0.112			
ΔPRTEE	2	16.50	12.75	14.75	14.50	14.00	10.50	0.961			
(vs week 0)	4	21.50	9.25	25.25	18.13	15.00	11.75	0.431			
	8	25.50	10.75	28.00	18.00	13.50	20.00	0.597			
	12	27.00	13.45	29.50	15.50	21.00	18.50	0.791			

	24	32.00	11.50	30.50	20.25	19.00	19.75	0.521			
	52	32.75	12.25	36.50	19.75	27.00	18.25	0.532			
	104	38.50	12.75	37.75	16.25	26.00	22.00	0.348			

**PROM**	**week**	**Genotypes of rs2253369**						***P* value**			
		**CC**		**CT**		**TT**					
		**Median**	**±QD**	**Median**	**±QD**	**Median**	**±QD**	**Kruskal-Wallis test**	**CC vs CT**	**CC vs TT**	**CT vs TT**
VAS	0	6.00	2.25	6.00	1.75	5.00	1.50	0.605			
	2	3.00	1.50	4.00	1.50	4.00	1.50	0.644			
	4	3.00	1.50	3.00	2.00	3.00	1.50	0.805			
	8	3.00	1.25	3.00	2.00	3.00	1.50	0.971			
	12	3.00	1.75	3.00	1.50	2.00	1.50	0.628			
	24	1.00	2.00	2.50	2.50	2.00	1.50	0.762			
	52	3.00	2.25	1.00	2.50	1.00	2.00	0.451			
	104	3.00	1.75	0.50	1.50	1.00	1.00	0.287			
ΔVAS (vs week 0)	2	1.00	1.75	1.00	1.50	1.00	1.50	0.973			
	4	2.25	2.00	2.00	2.00	2.00	1.50	0.853			
	8	1.25	2.25	2.00	2.00	3.00	2.00	0.835			
	12	2.00	2.50	3.00	2.00	2.50	2.00	0.784			
	24	2.00	3.00	2.50	2.00	3.00	2.00	0.830			
	52	0.50	3.50	4.00	2.50	3.00	2.00	0.283			
	104	3.50	3.50	4.25	2.50	4.00	1.50	0.697			
QDASH	0	56.81	10.91	50.00	13.64	52.27	12.64	0.180			
	2	38.64	8.52	42.05	18.18	38.64	15.91	0.913			
	4	38.64	7.39	31.82	15.91	36.36	14.77	0.793			
	8	35.23	17.61	29.55	19.32	36.36	17.05	0.711			
	12	31.82	24.43	27.27	14.77	29.55	18.18	0.856			
	24	15.91	29.55	25.00	23.86	27.27	20.45	0.879			
	52	26.14	26.14	15.91	23.86	20.45	21.59	0.706			
	104	32.95	22.16	5.68	17.05	14.77	17.05	0.180			
ΔQDASH (vs week 0)	2	13.63	11.93	4.31	14.86	6.81	13.13	0.539			
	4	19.31	10.79	11.36	19.32	10.22	13.64	0.586			
	8	30.68	17.05	13.63	20.45	14.77	18.18	0.282			
	12	30.68	17.50	22.72	19.98	15.90	16.48	0.531			
	24	43.18	20.45	21.36	19.32	19.31	17.05	0.509			
	52	26.13	19.77	27.27	23.87	20.45	18.18	0.604			
	104	32.95	23.87	30.68	25.01	29.55	15.90	0.554			

PRTEE	0	47.00	13.88	53.00	11.75	51.50	15.00	0.871			
	2	30.75	6.63	30.75	19.00	28.00	12.00	0.822			
	4	26.50	7.63	23.75	15.50	25.00	12.00	0.829			
	8	25.25	18.00	21.50	16.50	24.00	14.50	0.754			
	12	25.25	19.75	19.00	14.75	20.00	14.75	0.863			
	24	14.50	38.25	16.50	18.00	12.00	16.75	0.905			
	52	15.00	16.50	10.50	17.50	13.00	15.25	0.692			
	104	21.25	16.88	4.50	12.50	7.50	11.50	0.215			
ΔPRTEE (vs week 0)	2	12.00	6.38	15.00	15.25	16.00	9.75	0.465			
	4	14.50	13.63	21.25	15.25	21.75	12.63	0.727			
	8	15.00	18.50	29.00	18.25	25.25	12.38	0.428			
	12	10.00	20.75	29.50	15.50	28.00	13.98	0.326			
	24	19.00	21.00	33.00	19.50	27.75	16.50	0.766			
	52	23.00	18.88	38.00	19.50	30.25	14.25	0.357			
	104	27.50	19.13	38.25	17.25	38.50	11.50	0.318			

**PROM**	**week**	**Genotypes of rs35231764**						***P* value**			
		**AA**		**AG**		**GG**					
		**Median**	**±QD**	**Median**	**±QD**	**Median**	**±QD**	**Kruskal-Wallis test**	**AA vs AG**	**AA vs GG**	**AG vs GG**
VAS	0	5.00	2.00	6.00	1.50	4.00	1.50	0.004 *	0.018	0.843	0.026
	2	3.00	1.50	4.00	1.50	3.00	1.00	0.006 *	0.088	0.258	0.010
	4	3.00	1.50	4.00	1.50	2.00	1.25	0.036	0.722	0.169	0.034
	8	3.00	2.00	3.00	2.00	1.00	1.50	0.364			
	12	2.00	1.50	3.00	2.00	0.50	0.75	0.007 *	0.438	0.059	0.006
	24	2.00	2.00	2.00	2.00	3.00	3.00	0.900			
	52	2.00	2.00	2.00	2.00	0.00	1.00	0.042	0.429	0.362	0.060
	104	1.00	1.50	1.00	1.50	0.00	0.50	0.059			
ΔVAS (vs week 0)	2	1.00	1.50	1.00	1.50	1.50	1.25	0.748			
	4	2.00	2.00	2.00	1.50	1.50	1.75	0.301			
	8	2.00	2.00	3.00	2.00	3.00	2.50	0.135			
	12	3.00	1.50	3.00	2.00	3.50	1.75	0.752			
	24	2.00	1.50	4.00	2.00	2.00	3.00	0.048	0.097	1.000	0.202
	52	2.00	2.50	4.00	2.00	4.00	2.00	0.749			
	104	4.00	3.00	4.00	2.00	3.00	2.00	0.482			
QDASH	0	48.86	11.37	59.09	12.50	40.91	11.82	0.006 *	0.038	0.588	0.021
	2	36.36	11.36	50.00	19.32	23.86	12.50	0.017	0.010	0.658	0.011
	4	36.36	10.23	45.45	15.91	20.45	9.66	0.012	0.482	0.084	0.010

	8	29.55	17.05	36.36	21.59	26.14	20.45	0.341			
	12	27.27	17.05	34.09	19.32	22.73	13.64	0.076			
	24	25.00	23.86	25.00	18.75	31.82	26.14	0.705			
	52	17.05	23.86	25.00	22.73	13.64	13.64	0.181			
	104	9.09	20.45	15.91	20.45	4.55	28.41	0.307			
ΔQDASH (vs week 0)	2	6.81	12.50	4.09	15.91	6.82	12.00	0.669			
	4	11.36	15.34	13.63	14.64	12.27	13.63	0.732			
	8	13.63	21.59	17.04	18.18	13.63	21.59	0.568			
	12	18.18	17.05	15.91	19.32	22.50	18.18	0.955			
	24	21.59	19.88	20.45	19.66	2.27	23.86	0.049	0.623	0.245	0.049
	52	22.72	17.05	22.72	22.72	20.45	15.91	0.754			
	104	29.54	23.87	36.36	18.18	9.10	15.91	0.167			
PRTEE	0	49.50	10.50	63.00	14.00	38.75	16.25	0.001 *	0.002	1.000	0.032
	2	29.00	12.25	37.00	20.00	17.75	3.88	0.007 *	0.086	0.291	0.012
	4	24.50	12.75	30.25	15.50	13.75	8.50	0.092			
	8	23.00	14.00	27.00	16.25	13.75	11.75	0.352			
	12	19.50	14.75	24.00	19.50	9.75	12.38	0.972			
	24	13.50	17.25	15.00	15.50	19.50	25.50	0.593			
	52	11.50	15.75	13.00	19.50	6.00	11.00	0.137			
	104	5.75	14.00	9.00	11.75	1.00	12.75	0.303			
ΔPRTEE (vs week 0)	2	13.50	11.13	16.75	12.75	20.25	13.75	0.589			
	4	19.00	12.88	25.25	12.25	21.00	13.25	0.306			
	8	22.50	17.25	30.75	14.75	26.00	16.50	0.193			
	12	26.50	17.00	29.25	16.00	33.75	14.75	0.595			
	24	28.95	18.25	31.75	15.00	14.00	21.00	0.025	0.366	0.213	0.026
	52	33.50	19.25	33.00	16.50	29.50	14.75	0.600			
	104	37.00	19.25	40.00	14.00	38.50	13.25	0.110			
**PROM**	**week**	**Genotypes of rs1800012**						***P* value**			
		**AA**		**AC**		**CC**					
		**Median**	**±QD**	**Median**	**±QD**	**Median**	**±QD**	**Kruskal-Wallis test**	**AA vs AC**	**AA vs CC**	**AC vs CC**
VAS	0	5.00	1.25	7.00	1.50	5.00	1.50	0.027 *	0.486	1.000	0.025
	2	4.00	1.00	4.50	1.75	3.00	1.50	0.283			
	4	4.00	1.25	3.50	1.50	3.00	1.50	0.179			
	8	3.00	0.75	4.00	1.50	3.00	1.50	0.122			
	12	2.00	1.25	4.00	1.50	2.00	1.50	0.035	0.314	1.000	0.044
	24	2.50	1.00	4.00	2.50	2.00	2.00	0.053			

	52	1.50	1.50	3.00	2.50	1.00	2.00	0.436			
	104	1.00	0.75	1.00	1.50	1.00	1.50	0.971			
ΔVAS (vs week 0)	2	2.00	1.50	2.00	2.00	1.00	1.50	0.484			
	4	1.50	2.00	3.00	2.25	2.00	2.00	0.182			
	8	3.00	2.00	2.00	2.50	2.50	2.00	0.787			
	12	4.00	2.00	3.00	1.50	3.00	2.00	0.706			
	24	2.00	1.25	3.00	2.00	3.00	2.00	0.908			
	52	5.00	2.00	4.00	2.50	3.00	2.00	0.500			
	104	4.50	2.25	4.50	2.00	3.50	2.00	0.157			
QDASH	0	43.18	8.52	52.27	7.93	52.27	15.46	0.135			
	2	48.86	4.55	46.59	13.64	36.36	17.05	0.219			
	4	39.77	3.98	44.32	16.48	34.09	13.64	0.277			
	8	28.41	5.68	45.45	19.32	29.55	18.18	0.235			
	12	28.41	10.80	38.64	19.32	25.00	17.05	0.067			
	24	32.95	10.80	35.23	22.16	19.32	21.02	0.239			
	52	11.36	17.61	29.55	25.00	15.91	22.73	0.805			
	104	18.64	9.09	13.64	20.45	11.36	21.59	0.509			
ΔQDASH (vs week 0)	2	-3.41	13.63	10.22	11.36	6.81	14.78	0.073			
	4	1.13	9.66	11.36	14.76	15.68	14.78	0.108			
	8	11.36	13.64	11.36	15.44	17.04	19.32	0.559			
	12	19.31	14.77	15.90	15.86	21.59	19.32	0.261			
	24	11.36	18.18	19.31	18.18	25.00	20.32	0.282			
	52	22.72	18.18	22.73	22.73	21.59	19.39	0.794			
	104	18.18	14.21	36.36	22.73	30.68	22.73	0.241			
PRTEE	0	41.20	7.63	59.00	10.25	50.00	14.00	0.042	0.135	1.000	0.092
	2	36.00	9.50	40.00	15.00	25.00	16.00	0.104			
	4	28.00	4.88	31.25	20.75	23.00	12.75	0.310			
	8	20.25	6.25	33.50	19.75	21.00	14.50	0.180			
	12	17.00	8.63	24.00	19.75	16.50	14.00	0.101			
	24	20.50	12.00	27.75	20.50	11.75	15.63	0.230			
	52	10.50	12.25	20.00	19.25	11.00	13.25	0.633			
	104	15.25	8.13	6.50	14.00	7.00	12.75	0.493			
ΔPRTEE (vs week 0)	2	6.00	8.78	16.75	14.13	15.25	11.50	0.137			
	4	14.25	14.98	24.75	16.75	21.25	13.50	0.327			
	8	25.00	15.48	25.00	15.25	28.00	17.25	0.996			
	12	33.50	14.10	23.50	16.50	29.00	16.50	0.881			
	24	20.45	19.00	31.25	19.88	30.50	19.00	0.592			

	52	39.75	19.23	33.50	17.50	32.25	17.75	0.867			
	104	31.25	18.60	40.00	14.75	37.25	16.00	0.138			

**PROM**	**week**	**Genotypes of rs9898186**						***P* value**			
		**CC**		**CT**		**TT**					
		**Median**	**±QD**	**Median**	**±QD**	**Median**	**±QD**	**Kruskal-Wallis test**	**CC vs CT**	**CC vs TT**	**CT vs TT**
VAS	0	6.00	1.50	6.00	2.00	5.00	1.50	0.427			
	2	3.00	1.50	4.00	1.50	4.00	1.00	0.621			
	4	3.00	1.50	3.00	1.50	4.00	2.00	0.612			
	8	3.00	1.50	3.50	2.00	3.00	0.50	0.463			
	12	2.00	1.50	3.50	2.00	2.00	2.00	0.080			
	24	2.00	2.00	3.00	2.00	2.50	1.00	0.327			
	52	1.00	2.00	3.00	2.50	0.50	1.50	0.167			
	104	1.00	1.50	1.50	1.50	1.00	1.00	0.371			
ΔVAS (vs week 0)	2	1.00	1.50	1.00	2.00	2.00	1.50	0.662			
	4	2.00	2.00	2.00	2.00	2.50	1.50	0.814			
	8	3.00	2.00	2.00	2.00	4.00	1.50	0.462			
	12	3.00	2.00	2.00	2.00	4.50	2.00	0.362			
	24	3.00	2.00	2.50	1.50	3.00	1.50	0.895			
	52	3.00	2.00	3.00	2.00	5.00	2.00	0.310			
	104	4.00	2.00	4.00	2.50	5.00	2.50	0.686			
QDASH	0	51.14	15.91	52.27	6.36	43.18	8.00	0.202			
	2	36.36	17.05	45.45	15.91	46.59	7.95	0.549			
	4	31.82	13.64	43.18	15.91	37.50	6.82	0.227			
	8	32.95	19.32	37.50	20.45	27.27	7.95	0.414			
	12	26.14	17.05	34.09	19.32	27.27	11.36	0.143			
	24	23.86	22.73	25.00	20.45	31.82	13.64	0.739			
	52	15.91	23.30	28.41	23.30	7.95	17.05	0.443			
	104	11.36	21.59	13.64	21.02	18.18	11.36	0.894			
ΔQDASH (vs week 0)	2	4.54	14.77	9.09	12.50	-0.05	10.23	0.212			
	4	15.45	14.78	11.36	14.77	5.68	15.86	0.445			
	8	15.90	20.45	15.91	17.67	13.63	17.05	0.991			
	12	20.45	19.45	15.91	17.04	22.72	12.50	0.763			
	24	20.45	20.46	20.45	17.05	12.50	22.73	0.542			
	52	20.45	21.72	22.73	20.45	31.77	14.78	0.995			
	104	31.81	21.58	31.82	22.73	23.86	17.05	0.626			
PRTEE	0	51.25	15.25	55.00	11.00	41.20	8.25	0.227			

	2	26.00	15.25	37.00	18.50	33.50	8.00	0.423
	4	23.00	13.00	26.00	16.75	26.25	9.25	0.472
	8	21.75	16.00	25.50	18.63	18.00	6.25	0.382
	12	18.50	14.00	22.50	21.25	17.00	10.75	0.285
	24	13.75	16.75	18.50	18.50	17.50	9.75	0.823
	52	11.25	14.00	19.00	18.13	6.75	12.75	0.421
	104	7.25	12.50	7.00	14.38	9.00	10.00	0.885
ΔPRTEE (vs week 0)	2	15.00	12.50	16.50	13.50	10.50	8.80	0.404
	4	22.00	13.75	20.50	13.75	21.20	13.00	0.871
	8	27.50	18.00	25.25	14.63	28.75	13.45	0.845
	12	27.00	17.25	28.00	15.75	36.00	13.45	0.827
	24	28.50	19.25	31.25	17.75	32.20	19.00	0.831
	52	32.50	19.50	32.00	17.00	39.75	18.00	0.878
	104	38.50	16.25	38.00	16.38	35.75	19.75	0.735

Legend: *COL1A1*, gene for collagen, type I, alpha-1 chain; QD, Quartile Deviation; PROMs, patient-reported outcome measures; VAS, Visual Analog Scale; QDASH, quick version of Disabilities of the Arm, Shoulder and Hand score; PRTEE, Patient-Rated Tennis Elbow Evaluation; * differences significant after Hochberg correction for multiple comparisons.

Table S2. PROMs values in individuals with particular genotype variants of the *COL1A1* gene rs2249492 polymorphism in recessive/dominant model

CC vs CT/TT

PROM	week	CC		CT/TT		*P* Mann-Whitney U test
		median	± QD	median	± QD	
VAS	0	5.00	3.00	6.00	1.50	0.494
	2	3.00	1.00	4.00	1.50	0.017 *
	4	2.00	1.00	3.00	1.50	0.055
	8	3.00	1.50	3.00	2.00	0.439
	12	3.00	1.50	2.00	2.00	0.875
	24	1.00	2.00	2.00	2.00	0.492
	52	3.00	2.00	1.00	2.00	0.956
	104	1.00	1.50	1.00	1.50	0.928
ΔVAS (vs week 0)	2	1.00	2.00	1.00	1.50	0.444
	4	2.50	2.00	2.00	2.00	0.630
	8	2.00	2.00	3.00	2.00	0.769
	12	2.00	1.50	3.00	2.00	0.543
	24	2.00	2.00	3.00	2.00	0.608
	52	2.00	2.50	4.00	2.00	0.308
	104	2.50	2.25	4.00	2.00	0.451
QDASH	0	55.00	10.23	52.27	13.64	0.459
	2	31.82	11.36	43.18	16.25	0.060
	4	31.82	7.95	36.36	15.91	0.223
	8	25.00	14.77	34.09	17.05	0.231
	12	18.18	17.05	29.55	17.05	0.342
	24	11.36	24.43	25.00	21.02	0.624
	52	13.64	25.00	20.45	21.59	0.584
	104	13.64	21.59	13.64	20.45	0.636
ΔQDASH (vs week 0)	2	15.91	11.36	4.54	11.37	0.038
	4	20.45	11.37	9.09	15.91	0.079
	8	31.81	14.77	12.50	18.31	0.062
	12	27.27	15.89	17.08	17.05	0.168
	24	38.61	16.48	18.17	19.32	0.161
	52	34.09	20.23	21.59	21.02	0.375
	104	40.90	19.32	29.55	21.59	0.591
PRTEE	0	42.50	10.00	53.00	14.25	0.290
	2	27.00	12.50	31.50	17.00	0.172
	4	23.00	7.25	25.75	15.00	0.238
	8	19.50	11.50	24.00	16.00	0.428
	12	13.50	15.00	20.00	15.00	0.632
	24	8.00	20.25	15.25	16.75	0.648
	52	6.00	15.25	12.50	15.25	0.580
	104	10.00	14.00	7.00	12.75	0.768
ΔPRTEE (vs week 0)	2	17.50	10.00	14.50	11.75	0.604
	4	22.00	11.50	21.00	13.25	0.674
	8	29.50	12.00	25.50	16.75	0.856
	12	24.00	18.25	29.50	16.00	0.442
	24	35.00	17.63	30.50	19.25	0.684
	52	28.50	15.50	33.25	17.88	0.438
	104	37.00	14.75	38.50	15.50	0.242

TT vs CC/CT

PROM		TT		CC/CT		*P* Mann-Whitney U test
	week	median	± QD	median	± QD	
VAS	0	6.00	2.00	6.00	1.50	0.364
	2	3.00	1.00	4.00	2.00	0.867
	4	3.00	1.00	3.00	1.50	0.801
	8	3.00	1.50	3.00	2.00	0.819
	12	2.00	1.50	3.00	2.00	0.399
	24	2.00	2.00	2.00	2.00	0.464
	52	1.00	1.50	3.00	2.50	0.707
	104	1.00	1.00	1.00	1.50	0.680
ΔVAS (vs week 0)	2	2.00	1.50	1.00	1.50	0.817
	4	2.00	1.50	2.00	2.00	0.613
	8	3.00	1.50	2.00	2.00	0.968
	12	3.00	1.50	3.00	2.00	0.935
	24	3.00	2.00	2.00	2.00	0.607
	52	4.00	2.00	3.00	3.00	0.829
	104	4.00	2.00	4.00	2.50	0.720
QDASH	0	52.27	12.64	52.27	14.78	0.366
	2	40.91	13.64	38.64	15.91	0.700
	4	36.36	13.64	36.36	13.64	0.794
	8	36.36	21.59	29.55	18.18	0.524
	12	31.82	17.05	27.27	17.05	0.802
	24	27.27	20.45	25.00	20.46	0.867
	52	13.64	22.73	18.18	23.86	0.665
	104	15.91	17.05	9.09	21.59	0.463
ΔQDASH (vs week 0)	2	6.81	12.84	4.54	12.51	0.929
	4	9.09	14.77	13.64	15.91	0.671
	8	11.36	18.18	15.91	18.29	0.179
	12	13.64	18.18	22.72	14.78	0.317
	24	18.18	26.14	21.59	17.61	0.740
	52	20.45	21.59	25.00	19.32	0.355
	104	31.82	17.04	29.55	23.29	0.294
PRTEE	0	52.50	14.50	52.00	12.25	0.828
	2	24.50	12.50	34.00	17.50	0.599
	4	23.00	12.00	27.00	16.25	0.825
	8	22.00	17.00	22.00	15.75	0.944
	12	20.00	14.75	20.00	16.00	0.862
	24	13.50	17.25	15.00	16.50	0.714
	52	11.50	13.75	12.00	16.25	0.726
	104	7.50	11.50	7.00	14.00	0.602
ΔPRTEE (vs week 0)	2	19.50	11.25	13.50	12.00	0.345
	4	25.00	14.25	20.50	13.00	0.961
	8	25.50	14.45	26.75	16.50	0.904
	12	27.00	15.50	29.00	16.25	0.925
	24	27.40	19.50	31.50	18.13	0.950
	52	31.00	14.25	34.50	19.25	0.562
	104	39.00	15.75	37.50	17.00	0.909

Legend: *COL1A1*, Collagen, Type I, Alpha-1 gene; PROM, Patient-Reported Outcome Measure; PRTEE, Patient-Rated Tennis Elbow Evaluation; QD, Quartile Deviation; QDASH, quick version of Disabilities of the Arm, Shoulder and Hand score; VAS, Visual Analog Scale; * differences remaining significant after Hochberg correction for multiple comparisons.

Table S3. PROMs values in individuals with particular genotype variants of the *COL1A1* gene rs2586488 polymorphism in recessive/dominant model.

AA vs AG/GG

**PROM**		**AA**		**AG/GG**		***P* Mann-Whitney**
	week	median	± QD	median	± QD	**U test**
VAS	0	7.00	2.13	6.00	1.50	0.566
	2	3.00	1.50	4.00	1.50	0.521
	4	3.00	1.50	3.00	1.50	0.514
	8	3.00	1.00	3.00	2.00	0.645
	12	3.50	1.25	2.00	1.50	0.185
	24	2.00	2.00	2.00	2.00	0.648
	52	3.00	2.75	1.00	2.00	0.510
	104	2.00	1.50	1.00	1.50	0.470
ΔVAS (vs week 0)	2	1.25	1.75	1.00	1.50	0.566
	4	2.75	2.00	2.00	2.00	0.659
	8	1.75	2.50	2.00	2.00	0.854
	12	2.00	2.50	3.00	2.00	0.613
	24	3.00	3.00	3.00	2.00	0.948
	52	1.00	3.75	4.00	2.00	0.571
	104	4.00	3.50	4.00	2.00	0.709
QDASH	0	60.22	11.47	51.14	13.64	0.039 *
	2	36.36	10.23	40.91	17.05	0.832
	4	38.64	9.09	36.36	15.91	0.626
	8	35.23	17.05	31.82	19.32	0.736
	12	31.82	22.16	27.27	17.05	0.403
	24	31.82	29.55	25.00	20.45	0.428
	52	13.64	27.84	19.32	22.73	0.990
	104	25.00	21.59	12.50	19.32	0.595
ΔQDASH (vs week 0)	2	17.04	19.32	5.68	12.64	0.140
	4	19.31	15.34	11.36	15.91	0.188
	8	30.68	17.61	13.63	18.18	0.195
	12	30.68	17.49	18.18	17.05	0.523
	24	38.58	19.32	20.45	18.75	0.318
	52	38.64	20.34	20.45	20.45	0.240
	104	47.73	20.34	29.55	21.59	0.325
PRTEE	0	49.75	17.50	52.50	13.25	0.840
	2	31.50	6.63	29.50	17.50	0.615
	4	26.50	9.50	24.50	14.50	0.667
	8	25.25	15.63	22.00	16.00	0.358
	12	26.25	17.75	19.25	14.88	0.283
	24	19.50	36.75	14.00	17.00	0.466
	52	8.50	17.00	12.25	15.25	0.955
	104	14.75	15.13	7.00	12.63	0.527
ΔPRTEE (vs week 0)	2	13.25	8.63	15.25	12.75	0.609
	4	18.50	15.13	21.50	13.00	0.866
	8	15.00	20.88	27.50	15.75	0.378
	12	10.00	22.63	29.50	14.75	0.202
	24	19.00	23.25	30.75	18.25	0.586
	52	31.75	24.25	33.00	17.50	0.682
	104	33.25	25.13	38.00	16.50	0.654

GG vs AA/AG

PROM		GG		AA/AG		*P* Mann-Whitney U test
	week	median	± QD	median	± QD	
VAS	0	6.00	2.00	6.00	1.50	0.612
	2	4.00	1.50	4.00	2.00	0.342
	4	3.00	1.00	3.00	1.50	0.419
	8	3.00	1.50	3.00	2.00	0.415
	12	2.00	1.50	3.00	2.00	0.294
	24	2.00	1.50	2.00	2.00	0.306
	52	1.00	2.00	2.00	2.50	0.785
	104	1.00	1.00	1.00	1.50	0.823
ΔVAS (vs week 0)	2	2.00	1.50	1.00	1.50	0.411
	4	2.00	1.50	2.00	2.00	0.499
	8	3.00	1.50	2.00	2.00	0.552
	12	3.00	2.00	2.00	2.00	0.810
	24	3.00	1.50	2.00	1.75	0.262
	52	3.00	2.00	3.75	3.00	0.824
	104	4.00	2.00	4.00	2.50	0.648
QDASH	0	52.27	13.64	52.27	12.50	0.776
	2	40.91	15.91	38.64	15.91	0.937
	4	36.36	14.77	35.23	13.64	0.909
	8	36.36	18.18	29.55	18.18	0.545
	12	31.82	17.05	27.27	15.91	0.467
	24	27.27	20.45	25.00	20.46	0.988
	52	22.73	22.73	15.91	23.86	0.364
	104	15.91	20.45	9.09	20.45	0.083
ΔQDASH (vs week 0)	2	6.81	11.84	6.81	14.77	0.968
	4	11.36	12.50	13.64	16.91	0.762
	8	15.91	18.18	15.90	18.29	0.328
	12	15.90	17.05	22.73	15.91	0.352
	24	18.18	21.46	23.86	19.32	0.905
	52	18.17	21.59	28.36	18.19	0.223
	104	29.54	17.04	30.68	20.46	0.097
PRTEE	0	52.50	14.50	52.00	11.25	0.887
	2	27.50	11.00	33.50	17.75	0.279
	4	24.50	11.75	25.25	15.25	0.546
	8	24.00	13.75	22.00	19.00	0.613
	12	21.00	14.00	19.00	16.25	0.863
	24	13.50	17.25	16.00	17.00	0.559
	52	13.00	15.25	11.00	16.25	0.715
	104	8.00	12.25	7.00	14.00	0.267
ΔPRTEE (vs week 0)	2	17.50	9.75	13.50	12.50	0.110
	4	22.00	11.50	20.50	13.50	0.581
	8	27.50	14.25	25.25	17.50	0.685
	12	27.00	14.00	29.00	17.75	0.915
	24	30.50	18.50	31.00	18.50	0.658
	52	29.00	15.25	36.75	19.25	0.571
	104	38.50	15.75	37.75	16.75	0.816

Legend: *COL1A1*, Collagen, Type I, Alpha-1 gene; PROM, Patient-Reported Outcome Measure; PRTEE, Patient-Rated Tennis Elbow Evaluation; QD, Quartile Deviation; QDASH, quick version of Disabilities of the Arm, Shoulder and Hand score; VAS, Visual Analog Scale; * differences remaining significant after Hochberg correction for multiple comparisons.

Table S4. PROMs values in individuals with particular genotype variants of the *COL1A1* gene rs2075558 polymorphism in recessive/dominant model.

AA vs AC/CC

**PROM**		**AA**		**AC/CC**		***P* Mann-Whitney**
	week	median	± QD	median	± QD	**U test**
VAS	0	6.00	2.00	6.00	1.50	0.105
	2	3.00	2.00	4.00	1.50	0.815
	4	3.00	1.50	3.00	1.50	0.801
	8	4.00	2.00	3.00	2.00	0.992
	12	2.00	2.50	3.00	1.50	0.936
	24	2.00	2.00	2.00	2.00	0.844
	52	1.00	2.00	2.00	2.00	0.196
	104	0.00	1.00	1.00	1.50	0.023 *
ΔVAS (vs week 0)	2	2.00	1.50	1.00	1.50	0.177
	4	2.00	1.50	2.00	2.00	0.292
	8	3.00	2.00	2.00	2.00	0.222
	12	4.00	2.00	2.50	2.00	0.411
	24	3.00	1.50	3.00	2.00	0.220
	52	4.50	2.00	3.00	2.50	0.056
	104	5.00	2.00	3.75	2.50	0.013 *
QDASH	0	45.45	12.50	52.27	13.64	0.090
	2	40.91	17.05	38.64	16.08	0.392
	4	31.82	15.91	36.36	13.64	0.136
	8	34.09	22.73	31.82	15.91	0.506
	12	22.73	23.86	29.55	15.91	0.355
	24	15.91	15.91	27.27	21.59	0.236
	52	11.36	23.86	21.59	22.73	0.229
	104	5.68	11.36	19.77	21.59	0.089
ΔQDASH (vs week 0)	2	4.54	11.37	6.81	13.64	0.620
	4	6.82	13.64	13.64	17.04	0.542
	8	11.36	15.91	15.91	18.81	0.411
	12	18.17	18.18	18.18	16.55	0.467
	24	20.45	13.64	20.45	21.59	0.956
	52	22.72	14.77	20.45	21.73	0.766
	104	30.69	20.45	29.55	23.73	0.842
PRTEE	0	49.50	12.00	52.50	14.75	0.661
	2	33.50	15.75	28.00	16.75	0.862
	4	24.50	17.00	25.00	13.13	0.663
	8	24.00	16.75	22.00	15.75	0.533
	12	15.00	20.75	21.00	13.00	0.556
	24	11.00	17.00	18.00	16.50	0.308
	52	5.50	18.50	13.50	14.25	0.317
	104	1.75	7.50	9.75	14.00	0.069
ΔPRTEE (vs week 0)	2	16.50	12.75	14.50	11.75	0.841
	4	21.50	9.25	21.00	15.75	0.869
	8	25.50	10.75	26.50	18.13	0.980
	12	27.00	13.45	29.25	17.38	0.819
	24	32.00	11.50	28.25	19.75	0.475
	52	32.75	12.25	33.00	20.00	0.986
	104	38.50	12.75	37.00	18.25	0.487

CC vs AA/AC

PROM		CC		AA/AC		*P* Mann-Whitney U test
	week	median	± QD	median	± QD	
VAS	0	4.00	2.00	6.00	2.00	0.074
	2	3.00	1.00	4.00	1.50	0.263
	4	3.00	1.50	3.00	1.50	0.251
	8	2.00	2.00	3.00	2.00	0.344
	12	2.00	1.50	3.00	2.00	0.209
	24	2.00	1.50	2.00	2.00	0.644
	52	2.00	2.50	1.00	2.00	0.485
	104	1.00	1.50	1.00	1.50	0.697
ΔVAS (vs week 0)	2	1.00	2.00	1.00	1.50	0.541
	4	2.00	2.00	2.00	2.00	0.751
	8	2.00	1.50	3.00	2.00	0.401
	12	3.00	1.50	3.00	2.00	0.782
	24	2.00	1.50	3.00	2.00	0.397
	52	1.00	2.00	4.00	2.50	0.074
	104	2.00	3.00	4.00	2.00	0.082
QDASH	0	52.27	14.77	52.27	13.64	0.152
	2	36.36	13.64	42.05	16.08	0.244
	4	36.36	14.77	36.36	14.20	0.637
	8	25.00	19.32	34.09	17.05	0.354
	12	27.27	18.18	29.55	18.18	0.214
	24	22.73	19.32	25.00	21.59	0.879
	52	25.00	22.73	15.91	23.86	0.703
	104	6.82	21.59	13.64	20.45	0.777
ΔQDASH (vs week 0)	2	9.09	11.36	4.55	13.64	0.949
	4	11.36	12.50	13.63	14.78	0.368
	8	4.54	27.27	15.91	17.62	0.521
	12	4.54	23.86	18.18	14.78	0.781
	24	15.63	22.73	20.45	17.04	0.356
	52	18.17	25.00	22.73	19.32	0.328
	104	27.27	25.00	30.68	21.58	0.482
PRTEE	0	46.00	11.50	53.50	14.25	0.061
	2	27.00	12.75	31.00	17.00	0.199
	4	24.00	8.25	25.25	14.50	0.533
	8	15.50	17.00	24.00	15.75	0.298
	12	16.00	13.00	21.50	15.25	0.224
	24	16.00	15.50	14.50	17.75	0.742
	52	15.00	9.75	11.50	16.75	0.806
	104	5.00	16.25	7.50	12.50	0.709
ΔPRTEE (vs week 0)	2	14.00	10.50	15.00	13.75	0.805
	4	15.00	11.75	24.00	14.00	0.241
	8	13.50	20.00	28.00	15.50	0.330
	12	21.00	18.50	29.00	14.50	0.588
	24	19.00	19.75	31.25	17.38	0.289
	52	27.00	18.25	34.75	17.50	0.288
	104	26.00	22.00	38.25	15.00	0.155

Legend: *COL1A1*, Collagen, Type I, Alpha-1 gene; PROM, Patient-Reported Outcome Measure; PRTEE, Patient-Rated Tennis Elbow Evaluation; QD, Quartile Deviation; QDASH, quick version of Disabilities of the Arm, Shoulder and Hand score; VAS, Visual Analog Scale.

Table S5. PROMs values in individuals with particular genotype variants of the *COL1A1* gene rs2253369 polymorphism in recessive/dominant model.

CC vs CT/TT

**PROM**		**CC**		**CT/TT**		***P* Mann-Whitney**
	week	median	± QD	median	± QD	**U test**
VAS	0	6.00	2.25	6.00	1.63	0.898
	2	3.00	1.50	4.00	1.50	0.408
	4	3.00	1.50	3.00	1.50	0.514
	8	3.00	1.25	3.00	2.00	0.889
	12	3.00	1.75	2.00	1.50	0.491
	24	1.00	2.00	2.00	2.00	0.874
	52	3.00	2.25	1.00	2.00	0.208
	104	3.00	1.75	1.00	1.50	0.116
ΔVAS (vs week 0)	2	1.00	1.75	1.00	1.50	0.955
	4	2.25	2.00	2.00	2.00	0.939
	8	1.25	2.25	3.00	2.00	0.561
	12	2.00	2.50	3.00	2.00	0.526
	24	2.00	3.00	3.00	2.00	0.651
	52	0.50	3.50	4.00	2.00	0.150
	104	3.50	3.50	4.00	2.00	0.532
QDASH	0	56.81	10.91	51.14	13.64	0.071
	2	38.64	8.52	40.91	17.05	0.777
	4	38.64	7.39	36.36	15.91	0.500
	8	35.23	17.61	31.82	19.32	0.757
	12	31.82	24.43	27.27	16.48	0.692
	24	15.91	29.55	25.00	20.45	0.615
	52	26.14	26.14	18.18	22.73	0.582
	104	32.95	22.16	11.36	17.61	0.165
ΔQDASH (vs week 0)	2	13.63	11.93	5.68	13.63	0.279
	4	19.31	10.79	11.36	15.91	0.307
	8	30.68	17.05	13.63	18.18	0.230
	12	30.68	17.50	18.18	17.05	0.437
	24	43.18	20.45	20.45	18.75	0.247
	52	26.13	19.77	22.72	20.46	0.527
	104	32.95	23.87	29.55	22.72	0.952
PRTEE	0	47.00	13.88	52.75	13.63	0.698
	2	30.75	6.63	29.50	17.50	0.635
	4	26.50	7.63	24.50	14.50	0.544
	8	25.25	18.00	22.00	16.00	0.464
	12	25.25	19.75	19.75	14.88	0.590
	24	14.50	38.25	15.00	17.00	0.733
	52	15.00	16.50	11.75	15.75	0.520
	104	21.25	16.88	6.75	12.13	0.154
ΔPRTEE (vs week 0)	2	12.00	6.38	15.50	12.75	0.299
	4	14.50	13.63	21.50	13.75	0.440
	8	15.00	18.50	27.50	15.75	0.230
	12	10.00	20.75	29.50	15.00	0.163
	24	19.00	21.00	30.75	19.00	0.477
	52	23.00	18.88	33.50	17.50	0.279
	104	27.50	19.13	38.50	16.50	0.162

TT vs CC/CT

PROM		TT		CC/CT		*P* Mann-Whitney U test
	week	median	± QD	median	± QD	
VAS	0	5.00	1.50	6.00	1.75	0.367
	2	4.00	1.50	4.00	1.50	0.868
	4	3.00	1.50	3.00	2.00	0.803
	8	3.00	1.50	3.00	2.00	0.821
	12	2.00	1.50	3.00	2.00	0.404
	24	2.00	1.50	2.00	2.00	0.469
	52	1.00	2.00	2.00	2.50	0.714
	104	1.00	1.00	1.00	1.50	0.692
ΔVAS (vs week 0)	2	1.00	1.50	1.00	1.50	0.819
	4	2.00	1.50	2.00	2.00	0.616
	8	3.00	2.00	2.00	2.00	0.969
	12	2.50	2.00	3.00	2.00	0.936
	24	3.00	2.00	2.00	2.00	0.610
	52	3.00	2.00	4.00	2.50	0.829
	104	4.00	1.50	4.00	3.00	0.721
QDASH	0	52.27	12.64	52.27	13.64	0.366
	2	38.64	15.91	39.77	17.05	0.700
	4	36.36	14.77	36.36	13.64	0.794
	8	36.36	17.05	29.55	18.18	0.524
	12	29.55	18.18	27.27	15.91	0.802
	24	27.27	20.45	25.00	20.46	0.867
	52	20.45	21.59	15.91	25.00	0.667
	104	14.77	17.05	10.23	21.59	0.469
ΔQDASH (vs week 0)	2	6.81	13.13	6.81	14.77	0.929
	4	10.22	13.64	14.54	17.05	0.671
	8	14.77	18.18	18.17	20.45	0.179
	12	15.90	16.48	22.73	19.98	0.317
	24	19.31	17.05	22.72	21.59	0.740
	52	20.45	18.18	27.27	22.72	0.355
	104	29.55	15.90	30.68	25.01	0.294
PRTEE	0	51.50	15.00	52.50	12.25	0.828
	2	28.00	12.00	30.75	17.00	0.599
	4	25.00	12.00	24.25	15.00	0.825
	8	24.00	14.50	22.00	18.75	0.944
	12	20.00	14.75	20.00	16.25	0.862
	24	12.00	16.75	16.00	18.00	0.715
	52	13.00	15.25	11.00	17.25	0.728
	104	7.50	11.50	7.25	14.00	0.608
ΔPRTEE (vs week 0)	2	16.00	9.75	13.75	14.50	0.345
	4	21.75	12.63	20.75	14.75	0.961
	8	25.25	12.38	28.00	18.00	0.904
	12	28.00	13.98	29.00	18.75	0.925
	24	27.75	16.50	31.50	18.50	0.950
	52	30.25	14.25	36.00	20.00	0.562
	104	38.50	11.50	37.75	18.75	0.909

Legend: *COL1A1*, Collagen, Type I, Alpha-1 gene; PROM, Patient-Reported Outcome Measure; PRTEE, Patient-Rated Tennis Elbow Evaluation; QD, Quartile Deviation; QDASH, quick version of Disabilities of the Arm, Shoulder and Hand score; VAS, Visual Analog Scale.

Table S6. PROMs values in individuals with particular genotype variants of the *COL1A1* gene rs35231764 polymorphism in recessive/dominant model.

AA vs AG/GG

**PROM**		**AA**		**AG/GG**		***P* Mann-Whitney**
	week	median	± QD	median	± QD	**U test**
VAS	0	5.00	2.00	6.00	2.00	0.048
	2	3.00	1.50	4.00	1.50	0.204
	4	3.00	1.50	3.00	1.50	0.733
	8	3.00	2.00	3.00	2.00	0.848
	12	2.00	1.50	3.00	2.00	0.665
	24	2.00	2.00	2.00	2.00	0.655
	52	2.00	2.00	1.00	2.50	0.459
	104	1.00	1.50	1.00	1.50	0.770
ΔVAS (vs week 0)	2	1.00	1.50	1.00	1.50	0.461
	4	2.00	2.00	2.00	1.50	0.167
	8	2.00	2.00	3.00	1.50	0.078
	12	3.00	1.50	3.00	2.00	0.488
	24	2.00	1.50	3.00	2.50	0.104
	52	2.00	2.50	4.00	2.00	0.464
	104	4.00	3.00	4.00	2.00	0.306
QDASH	0	48.86	11.37	53.41	14.77	0.092
	2	36.36	11.36	44.32	21.59	0.037
	4	36.36	10.23	37.50	15.91	0.661
	8	29.55	17.05	35.23	22.73	0.396
	12	27.27	17.05	29.55	19.32	0.282
	24	25.00	23.86	25.00	19.32	0.414
	52	17.05	23.86	19.32	22.73	0.211
	104	9.09	20.45	15.91	20.46	0.174
ΔQDASH (vs week 0)	2	6.81	12.50	4.54	14.77	0.423
	4	11.36	15.34	13.63	14.77	0.442
	8	13.63	21.59	15.91	17.05	0.809
	12	18.18	17.05	15.94	18.18	0.843
	24	21.59	19.88	20.45	20.46	0.624
	52	22.72	17.05	21.59	22.16	0.893
	104	29.54	23.87	31.82	20.45	0.750
PRTEE	0	49.50	10.50	56.00	16.00	0.008 *
	2	29.00	12.25	30.00	18.00	0.194
	4	24.50	12.75	24.75	13.75	0.635
	8	23.00	14.00	22.00	16.00	0.911
	12	19.50	14.75	21.50	18.00	0.416
	24	13.50	17.25	15.00	17.75	0.310
	52	11.50	15.75	12.25	16.63	0.324
	104	5.75	14.00	8.25	12.00	0.246
ΔPRTEE (vs week 0)	2	13.50	11.13	17.25	13.50	0.363
	4	19.00	12.88	24.50	13.50	0.182
	8	22.50	17.25	29.75	14.50	0.108
	12	26.50	17.00	29.50	15.50	0.312
	24	28.95	18.25	31.00	18.25	0.474
	52	33.50	19.25	32.75	15.25	0.437
	104	37.00	19.25	39.00	13.75	0.097

GG vs AA/AG

PROM	week	GG median	GG ± QD	AA/AG median	AA/AG ± QD	*P* Mann-Whitney U test
VAS	0	4.00	1.50	6.00	2.00	0.068
	2	3.00	1.00	4.00	1.50	0.019
	4	2.00	1.25	3.00	1.50	0.022
	8	1.00	1.50	3.00	2.00	0.165
	12	0.50	0.75	3.00	2.00	0.005 *
	24	3.00	3.00	2.00	2.00	0.931
	52	0.00	1.00	2.00	2.00	0.046
	104	0.00	0.50	1.00	1.50	0.035
ΔVAS (vs week 0)	2	1.50	1.25	1.00	1.50	0.702
	4	1.50	1.75	2.00	2.00	0.868
	8	3.00	2.50	2.00	2.00	0.782
	12	3.50	1.75	3.00	2.00	0.619
	24	2.00	3.00	3.00	2.00	0.234
	52	4.00	2.00	3.25	2.00	0.946
	104	3.00	2.00	4.00	2.50	0.801
QDASH	0	40.91	11.82	52.27	12.50	0.049
	2	23.86	12.50	40.91	15.91	0.043
	4	20.45	9.66	36.36	14.77	0.009 *
	8	26.14	20.45	34.09	18.75	0.403
	12	22.73	13.64	29.55	18.75	0.129
	24	31.82	26.14	25.00	21.59	0.682
	52	13.64	13.64	20.45	23.86	0.390
	104	4.55	28.41	13.64	20.45	0.862
ΔQDASH (vs week 0)	2	6.82	12.00	6.81	13.64	0.920
	4	12.27	13.63	12.50	15.91	0.923
	8	13.63	21.59	15.90	18.19	0.374
	12	22.50	18.18	18.18	17.05	0.886
	24	2.27	23.86	20.45	19.32	0.036
	52	20.45	15.91	22.72	21.59	0.516
	104	9.10	15.91	30.68	21.59	0.099
PRTEE	0	38.75	16.25	52.75	13.38	0.132
	2	17.75	3.88	32.50	16.75	0.023
	4	13.75	8.50	25.50	14.25	0.065
	8	13.75	11.75	24.00	15.88	0.189
	12	9.75	12.38	21.25	15.63	0.109
	24	19.50	25.50	14.50	16.50	0.692
	52	6.00	11.00	12.50	15.75	0.192
	104	1.00	12.75	7.50	12.75	0.589
ΔPRTEE (vs week 0)	2	20.25	13.75	14.50	11.50	0.452
	4	21.00	13.25	21.50	13.50	0.788
	8	26.00	16.50	25.50	16.75	0.811
	12	33.75	14.75	28.00	17.25	0.808
	24	14.00	21.00	31.25	19.63	0.025
	52	29.50	14.75	33.25	19.00	0.726
	104	38.50	13.25	38.00	16.75	0.512

Legend: *COL1A1*, Collagen, Type I, Alpha-1 gene; PROM, Patient-Reported Outcome Measure; PRTEE, Patient-Rated Tennis Elbow Evaluation; QD, Quartile Deviation; QDASH, quick version of Disabilities of the Arm, Shoulder and Hand score; VAS, Visual Analog Scale.

Table S7. PROMs values in individuals with particular genotype variants of the *COL1A1* gene rs1800012 polymorphism in recessive/dominant model.

AA vs AC/CC

**PROM**		**AA**		**AC/CC**		***P* Mann-Whitney**
	week	median	± QD	median	± QD	**U test**
VAS	0	5.00	1.25	6.00	1.63	0.665
	2	4.00	1.00	4.00	1.50	0.487
	4	4.00	1.25	3.00	1.50	0.106
	8	3.00	0.75	3.00	2.00	0.579
	12	2.00	1.25	3.00	2.00	0.449
	24	2.50	1.00	2.00	2.00	0.570
	52	1.50	1.50	2.00	2.50	0.454
	104	1.00	0.75	1.00	1.50	0.992
ΔVAS (vs week 0)	2	2.00	1.50	1.00	1.50	0.706
	4	1.50	2.00	2.00	2.00	0.303
	8	3.00	2.00	2.00	2.00	0.582
	12	4.00	2.00	3.00	2.00	0.436
	24	2.00	1.25	3.00	2.00	0.664
	52	5.00	2.00	3.00	2.00	0.468
	104	4.50	2.25	4.00	2.00	0.954
QDASH	0	43.18	8.52	52.27	13.64	0.074
	2	48.86	4.55	38.64	17.05	0.211
	4	39.77	3.98	36.36	15.91	0.495
	8	28.41	5.68	34.09	19.32	0.570
	12	28.41	10.80	28.41	18.75	0.815
	24	32.95	10.80	25.00	21.59	0.671
	52	11.36	17.61	19.32	23.86	0.686
	104	18.64	9.09	12.50	21.59	0.248
ΔQDASH (vs week 0)	2	-3.41	13.63	6.82	13.64	0.025 *
	4	1.13	9.66	13.64	14.77	0.037
	8	11.36	13.64	15.91	18.29	0.504
	12	19.31	14.77	18.18	19.32	0.425
	24	11.36	18.18	20.45	20.32	0.133
	52	22.72	18.18	22.72	21.59	0.571
	104	18.18	14.21	31.81	22.73	0.136
PRTEE	0	41.20	7.63	52.75	14.38	0.201
	2	36.00	9.50	28.00	16.75	0.277
	4	28.00	4.88	24.00	15.00	0.408
	8	20.25	6.25	22.00	16.63	0.480
	12	17.00	8.63	20.50	16.00	0.570
	24	20.50	12.00	14.00	17.38	0.490
	52	10.50	12.25	12.25	15.50	0.969
	104	15.25	8.13	6.75	13.38	0.236
ΔPRTEE (vs week 0)	2	6.00	8.78	15.50	12.50	0.049
	4	14.25	14.98	21.50	13.75	0.183
	8	25.00	15.48	27.50	16.75	0.977
	12	33.50	14.10	28.00	16.75	1.000
	24	20.45	19.00	31.00	19.25	0.328
	52	39.75	19.23	32.50	18.25	0.903
	104	31.25	18.60	38.00	16.50	0.222

CC vs AA/AC

PROM		CC		AA/AC		*P* Mann-Whitney U test
	week	median	± QD	median	± QD	
VAS	0	5.00	1.50	7.00	1.50	0.023 *
	2	3.00	1.50	4.00	1.00	0.117
	4	3.00	1.50	4.00	1.75	0.149
	8	3.00	1.50	4.00	1.50	0.111
	12	2.00	1.50	4.00	1.50	0.048
	24	2.00	2.00	3.00	2.25	0.018 *
	52	1.00	2.00	2.00	2.50	0.505
	104	1.00	1.50	1.00	1.00	0.828
ΔVAS (vs week 0)	2	1.00	1.50	2.00	1.75	0.371
	4	2.00	2.00	3.00	2.00	0.315
	8	2.50	2.00	2.00	2.50	0.558
	12	3.00	2.00	3.00	2.00	0.980
	24	3.00	2.00	2.50	1.75	0.891
	52	3.00	2.00	4.00	2.50	0.261
	104	3.50	2.00	4.50	2.50	0.072
QDASH	0	52.27	15.46	52.27	6.82	0.889
	2	36.36	17.05	47.73	10.23	0.111
	4	34.09	13.64	42.05	13.07	0.110
	8	29.55	18.18	38.64	17.05	0.206
	12	25.00	17.05	34.09	17.05	0.041
	24	19.32	21.02	34.09	21.59	0.095
	52	15.91	22.73	29.55	22.73	0.750
	104	11.36	21.59	18.18	17.05	0.631
ΔQDASH (vs week 0)	2	6.81	14.78	6.81	10.23	0.621
	4	15.68	14.78	6.81	13.07	0.299
	8	17.04	19.32	11.36	15.44	0.302
	12	21.59	19.32	15.90	15.86	0.105
	24	25.00	20.32	17.04	19.87	0.298
	52	21.59	19.39	22.73	21.59	0.580
	104	30.68	22.73	29.55	22.73	0.874
PRTEE	0	50.00	14.00	55.00	13.05	0.130
	2	25.00	16.00	38.50	14.00	0.035
	4	23.00	12.75	28.00	16.38	0.133
	8	21.00	14.50	27.00	16.25	0.190
	12	16.50	14.00	24.00	18.25	0.091
	24	11.75	15.63	26.00	18.75	0.088
	52	11.00	13.25	19.50	18.25	0.389
	104	7.00	12.75	7.50	12.75	0.683
ΔPRTEE (vs week 0)	2	15.25	11.50	14.50	12.38	0.609
	4	21.25	13.50	21.50	13.75	0.920
	8	28.00	17.25	25.00	15.25	0.954
	12	29.00	16.50	24.00	15.25	0.644
	24	30.50	19.00	31.00	18.63	0.913
	52	32.25	17.75	37.50	17.50	0.667
	104	37.25	16.00	39.00	15.00	0.320

Legend: *COL1A1*, Collagen, Type I, Alpha-1 gene; PROM, Patient-Reported Outcome Measure; PRTEE, Patient-Rated Tennis Elbow Evaluation; QD, Quartile Deviation; QDASH, quick version of Disabilities of the Arm, Shoulder and Hand score; VAS, Visual Analog Scale.

Table S8. PROMs values in individuals with particular genotype variants of the *COL1A1* gene rs9898186 polymorphism in recessive/dominant model.

CC vs CT/TT

**PROM**		**CC**		**CT/TT**		***P* Mann-Whitney U test**
	week	median	± QD	median	± QD	
VAS	0	6.00	1.50	6.00	2.00	0.220
	2	3.00	1.50	4.00	1.50	0.604
	4	3.00	1.50	3.00	1.50	0.432
	8	3.00	1.50	3.00	2.00	0.593
	12	2.00	1.50	3.00	2.00	0.117
	24	2.00	2.00	3.00	2.00	0.152
	52	1.00	2.00	2.00	2.50	0.534
	104	1.00	1.50	1.00	1.50	0.371
ΔVAS (vs week 0)	2	1.00	1.50	2.00	1.50	0.410
	4	2.00	2.00	2.00	2.00	0.573
	8	3.00	2.00	2.00	2.50	0.660
	12	3.00	2.00	3.00	2.00	0.953
	24	3.00	2.00	2.50	1.75	0.803
	52	3.00	2.00	3.75	2.50	0.674
	104	4.00	2.00	4.00	2.50	0.548
QDASH	0	51.14	15.91	52.27	7.96	0.906
	2	36.36	17.05	45.45	13.64	0.276
	4	31.82	13.64	40.91	13.64	0.113
	8	32.95	19.32	32.95	18.18	0.848
	12	26.14	17.05	30.68	18.18	0.147
	24	23.86	22.73	29.55	20.45	0.468
	52	15.91	23.30	22.73	23.86	0.861
	104	11.36	21.59	15.91	19.32	0.648
ΔQDASH (vs week 0)	2	4.54	14.77	6.81	10.23	0.902
	4	15.45	14.78	9.09	13.63	0.486
	8	15.90	20.45	15.91	18.18	0.914
	12	20.45	19.45	17.04	15.91	0.466
	24	20.45	20.46	20.45	16.48	0.902
	52	20.45	21.72	25.00	20.45	0.966
	104	31.81	21.58	29.55	22.73	0.992
PRTEE	0	51.25	15.25	54.00	12.25	0.397
	2	26.00	15.25	36.50	16.25	0.254
	4	23.00	13.00	26.00	14.00	0.235
	8	21.75	16.00	23.00	18.00	0.691
	12	18.50	14.00	21.50	17.75	0.338
	24	13.75	16.75	17.50	18.00	0.541
	52	11.25	14.00	16.50	15.75	0.587
	104	7.25	12.50	7.25	14.00	0.643
ΔPRTEE (vs week 0)	2	15.00	12.50	14.50	11.25	0.822
	4	22.00	13.75	20.50	12.75	0.894
	8	27.50	18.00	25.50	15.25	0.835
	12	27.00	17.25	30.25	14.00	0.890
	24	28.50	19.25	31.25	16.88	0.806
	52	32.50	19.50	34.25	17.50	0.713
	104	38.50	16.25	37.75	16.50	0.844

TT vs CC/CT

PROM		TT		CC/CT		*P* Mann-Whitney U test
	week	median	± QD	median	± QD	
VAS	0	5.00	1.50	6.00	1.50	0.969
	2	4.00	1.00	4.00	1.50	0.342
	4	4.00	2.00	3.00	1.50	0.408
	8	3.00	0.50	3.00	2.00	0.392
	12	2.00	2.00	3.00	2.00	0.343
	24	2.50	1.00	2.00	2.00	0.861
	52	0.50	1.50	2.00	2.50	0.146
	104	1.00	1.00	1.00	1.50	0.503
ΔVAS (vs week 0)	2	2.00	1.50	1.00	1.50	0.972
	4	2.50	1.50	2.00	2.00	0.944
	8	4.00	1.50	2.00	2.00	0.216
	12	4.50	2.00	3.00	2.00	0.189
	24	3.00	1.50	3.00	2.00	0.780
	52	5.00	2.00	3.00	2.00	0.128
	104	5.00	2.50	4.00	2.00	0.433
QDASH	0	43.18	8.00	52.27	13.64	0.103
	2	46.59	7.95	38.64	17.05	0.661
	4	37.50	6.82	36.36	15.91	0.945
	8	27.27	7.95	34.09	19.32	0.245
	12	27.27	11.36	29.55	19.32	0.454
	24	31.82	13.64	25.00	21.59	1.000
	52	7.95	17.05	20.45	23.86	0.213
	104	18.18	11.36	13.64	21.59	0.816
ΔQDASH (vs week 0)	2	-0.05	10.23	6.82	13.64	0.110
	4	5.68	15.86	13.63	14.77	0.216
	8	13.63	17.05	15.91	18.29	0.976
	12	22.72	12.50	18.18	18.18	0.856
	24	12.50	22.73	20.45	20.32	0.285
	52	31.77	14.78	20.45	21.59	0.951
	104	23.86	17.05	31.81	22.73	0.362
PRTEE	0	41.20	8.25	52.75	14.25	0.267
	2	33.50	8.00	28.00	16.75	0.420
	4	26.25	9.25	24.50	14.75	0.893
	8	18.00	6.25	23.00	16.50	0.269
	12	17.00	10.75	20.50	16.50	0.397
	24	17.50	9.75	14.00	17.50	0.752
	52	6.75	12.75	12.75	15.88	0.352
	104	9.00	10.00	7.25	14.00	0.776
ΔPRTEE (vs week 0)	2	10.50	8.80	15.25	12.50	0.187
	4	21.20	13.00	21.50	13.75	0.603
	8	28.75	13.45	25.50	16.75	0.564
	12	36.00	13.45	27.00	16.50	0.609
	24	32.20	19.00	30.50	19.00	0.667
	52	39.75	18.00	32.00	18.50	0.650
	104	35.75	19.75	38.00	16.50	0.520

Legend: *COL1A1*, Collagen, Type I, Alpha-1 gene; PROM, Patient-Reported Outcome Measure; PRTEE, Patient-Rated Tennis Elbow Evaluation; QD, Quartile Deviation; QDASH, quick version of Disabilities of the Arm, Shoulder and Hand score; VAS, Visual Analog Scale.

Table S9. The frequencies (%) and medians (± QD) of basic demographic and clinical characteristics of patients in relation to genotypes of the *COL1A1* gene polymorphisms.

		**Genotypes**						***p***		
**rs2249492**	**Parameter**	**CC**		**CT**		**TT**		**Additive model**	**Recessive/dominant model**	
		**Median**	**±QD**	**Median**	**±QD**	**Median**	**±QD**	CC vs (CT vs TT)	CC vs CT/TT	TT vs CC/CT
	BMI, median ± QD	26.51	2.00	25.28	1.59	26.06	2.64	0.959	0.937	0.815
	Cigarettes n/day, median ± QD	0.00	0.00	0.00	0.00	0.00	0.00	0.243	0.305	0.111
	Alcohol units/ week, median ± QD	0.50	1.00	0.25	1.00	1.00	2.25	0.570	0.886	0.299
		**n**	**%**	**n**	**%**	**n**	**%**	CC vs (CT vs TT)	CC vs CT/TT	TT vs CC/CT
	Cigarette smoking, n (%)	5	23.81	12	19.35	5	10.22	0.277	0.197	0.523
	Hypertension, n (%)	3	14.29	7	11.29	8	16.33	0.741	0.668	0.801
	Diabetes mellitus, n (%)	0	0.00	3	4.84	1	2.04	0.470	0.525	0.850
	Gout, n (%)	1	4.76	3	4.84	4	8.16	0.739	0.689	0.821
	Physical therapy, n (%)	7	33.33	24	38.71	25	51.02	0.280	0.125	0.358
	Manual therapy, n (%)	6	28.57	16	25.81	15	30.61	0.853	0.612	0.952
	NSAIDs, n (%)	11	52.38	17	27.42	13	26.53	0.070	0.387	0.021
	Vit. B12 suppl., n (%)	1	4.76	15	24.19	12	24.49	0.132	0.479	0.085
		**Genotypes**						***p***		
**rs2586488**	**Parameter**	**AA**		**AG**		**GG**		**Additive model**	**Recessive/dominant model**	
		**Median**	**±QD**	**Median**	**±QD**	**Median**	**±QD**	AA vs (AG vs GG)	AA vs AG/GG	GG vs AA/AG
	BMI, median ± QD	26.61	1.81	25.18	1.84	26.44	2.80	0.436	0.715	0.276
	Cigarettes n/day, median ± QD	0.00	6.25	0.00	0.00	0.00	0.00	0.150	0.246	0.829
	Alcohol units/ week, median ± QD	0.00	1.00	1.00	1.00	1.00	2.25	0.353	0.270	0.266
		**n**	**%**	**n**	**%**	**n**	**%**	AA vs (AG vs GG)	AA vs AG/GG	GG vs AA/AG
	Cigarette smoking, n (%)	4	33.33	8	12.70	10	17.54	0.207	0.223	0.814
	Hypertension, n (%)	3	25.00	7	11.11	8	14.04	0.435	0.446	0.907
	Diabetes mellitus, n (%)	0	0.00	3	4.76	1	1.75	0.513	0.810	0.816
	Gout, n (%)	1	8.33	3	4.76	4	7.02	0.824	0.773	0.973
	Physical therapy, n (%)	5	41.67	23	36.51	28	49.12	0.377	0.802	0.175
	Manual therapy, n (%)	3	25.00	17	26.98	17	29.82	0.914	0.927	0.689
	NSAIDs, n (%)	5	41.67	21	33.33	15	26.32	0.501	0.613	0.304
	Vit. B12 suppl., n (%)	0	0.00	14	22.22	14	24.56	0.161	0.130	0.412
		**Genotypes**						***p***		
**rs2075558**	**Parameter**	**AA**		**AC**		**CC**		**Additive model**	**Recessive/dominant model**	
		**Median**	**±QD**	**Median**	**±QD**	**Median**	**±QD**	AA vs (AC vs CC)	AA vs AC/CC	CC vs AA/AC
	BMI, median ± QD	25.08	2.45	25.95	2.40	24.86	1.37	0.240	0.881	0.121
	Cigarettes n/day, median ± QD	0.00	0.00	0.00	0.00	0.00	5.00	0.045	0.066	0.165
	Alcohol units/ week, median ± QD	2.00	2.50	0.00	1.00	1.00	1.00	0.025	0.012 *	0.846

		n	%	n	%	n	%	AA vs (AC vs CC)	AA vs AC/CC	CC vs AA/AC
	Cigarette smoking, n (%)	3	7.69	12	17.14	7	30.43	0.069	0.125	0.100
	Hypertension, n (%)	4	10.26	11	15.71	3	13.04	0.725	0.649	0.808
	Diabetes mellitus, n (%)	1	2.56	3	4.29	0	0.00	0.570	0.723	0.792
	Gout, n (%)	3	7.69	5	7.14	0	0.00	0.404	0.913	0.390
	Physical therapy, n (%)	14	35.90	33	47.14	9	39.13	0.492	0.326	0.904
	Manual therapy, n (%)	10	25.64	21	30.00	6	26.09	0.866	0.692	0.978
	NSAIDs, n (%)	8	20.51	23	32.86	10	43.48	0.150	0.090	0.157
	Vit. B12 suppl., n (%)	7	17.95	17	24.29	4	17.39	0.655	0.553	0.831
**rs2253369**	**Parameter**	**Genotypes**						***p***		
		**CC**		**CT**		**TT**		**Additive model**	**Recessive/dominant model**	
		**Median**	**±QD**	**Median**	**±QD**	**Median**	**±QD**	CC vs (CT vs TT)	CC vs CT/TT	TT vs CC/CT
	BMI, median ± QD	26.61	1.86	25.28	1.84	25.47	3.13	0.563	0.625	0.444
	Cigarettes n/day, median ± QD	0.00	6.25	0.00	0.00	0.00	0.00	0.050	0.246	0.187
	Alcohol units/ week, median ± QD	0.00	1.00	0.00	1.00	1.00	2.50	0.187	0.541	0.068
		**n**	%	**n**	%	**n**	%	CC vs (CT vs TT)	CC vs CT/TT	TT vs CC/CT
	Cigarette smoking, n (%)	4	33.33	5	8.47	13	21.31	0.045	0.223	0.274
	Hypertension, n (%)	3	25.00	6	10.17	9	14.75	0.371	0.446	0.729
	Diabetes mellitus, n (%)	0	0.00	3	5.08	1	1.64	0.444	0.810	0.723
	Gout, n (%)	1	8.33	2	3.39	5	8.20	0.512	0.773	0.557
	Physical therapy, n (%)	4	33.33	22	37.29	30	49.18	0.336	0.717	0.146
	Manual therapy, n (%)	3	25.00	14	23.73	20	32.79	0.527	0.927	0.259
	NSAIDs, n (%)	6	50.00	19	32.20	16	26.23	0.258	0.246	0.266
	Vit. B12 suppl., n (%)	0	0.00	11	8.33	17	27.87	0.079	0.130	0.083
**rs35231764**	**Parameter**	**Genotypes**						***p***		
		**AA**		**AG**		**GG**		**Additive model**	**Recessive/dominant model**	
		**Median**	**±QD**	**Median**	**±QD**	**Median**	**±QD**	AA vs (AG vs GG)	AA vs AG/GG	GG vs AA/AG
	BMI, median ± QD	25.28	2.65	25.99	2.38	25.67	1.36	0.574	0.634	0.469
	Cigarettes n/day, median ± QD	0.00	0.00	0.00	0.00	0.00	0.00	0.130	0.302	0.092
	Alcohol units/ week, median ± QD	0.50	1.00	1.00	1.00	4.50	3.75	0.090	0.037	0.358
		**n**	%	**n**	%	**n**	%	AA vs (AG vs GG)	AA vs AG/GG	GG vs AA/AG
	Cigarette smoking, n (%)	15	21.43	7	14.00	0	0.00	0.150	0.184	0.223
	Hypertension, n (%)	7	10.00	8	16.00	3	25.00	0.311	0.299	0.466
	Diabetes mellitus, n (%)	3	4.29	1	2.00	0	0.00	0.628	0.700	0.810
	Gout, n (%)	5	7.14	3	6.00	0	0.00	0.632	0.850	0.773
	Physical therapy, n (%)	26	37.14	50	50.00	5	41.67	0.372	0.192	0.802
	Manual therapy, n (%)	18	25.71	17	34.00	2	16.67	0.399	0.529	0.560
	NSAIDs, n (%)	25	35.71	13	26.00	3	25.00	0.470	0.220	0.882
	Vit. B12 suppl., n (%)	10	14.29	15	30.00	3	25.00	0.109	0.039	0.973

rs1800012	Parameter	Genotypes						*p*		
		AA		AC		CC		Additive model	Recessive/dominant model	
		**Median**	**±QD**	**Median**	**±QD**	**Median**	**±QD**	AA vs (AC vs CC)	AA vs AC/CC	CC vs AA/AC
	BMI, median ± QD	24.17	6.74	26.45	2.98	25.83	1.83	0.184	0.634	0.150
	Cigarettes n/day, median ± QD	0.00	0.00	0.00	0.00	0.00	0.00	0.372	0.826	0.164
	Alcohol units/ week, median ± QD	0.50	0.75	1.00	1.00	1.00	2.00	0.803	0.552	0.906
		**n**	**%**	**n**	**%**	**n**	**%**	AA vs (AC vs CC)	AA vs AC/CC	CC vs AA/AC
	Cigarette smoking, n (%)	1	12.50	3	9.09	18	19.78	0.350	0.870	0.239
	Hypertension, n (%)	0	0.00	3	9.09	15	16.48	0.291	0.530	0.252
	Diabetes mellitus, n (%)	0	0.00	3	9.09	1	1.10	0.063	0.584	0.168
	Gout, n (%)	0	0.00	5	15.15	3	3.30	0.038	0.981	0.112
	Physical therapy, n (%)	3	37.50	16	48.48	37	40.66	0.708	0.938	0.541
	Manual therapy, n (%)	3	37.50	9	27.27	25	27.47	0.827	0.834	0.832
	NSAIDs, n (%)	2	25.00	9	27.27	30	32.97	0.774	0.990	0.481
	Vit. B12 suppl., n (%)	1	12.50	7	21.21	20	21.98	0.821	0.860	0.928
**rs9898186**	**Parameter**	**Genotypes**						***p***		
		**CC**		**CT**		**TT**		**Additive model**	**Recessive/dominant model**	
		**Median**	**±QD**	**Median**	**±QD**	**Median**	**±QD**	CC vs (CT vs TT)	CC vs CT/TT	TT vs CC/CT
	BMI, median ± QD	25.47	1.56	26.74	2.39	26.07	6.33	0.053	0.020	0.862
	Cigarettes n/day, median ± QD	0.00	0.00	0.00	0.00	0.00	0.00	0.871	0.740	0.869
	Alcohol units/ week, median ± QD	1.00	2.00	1.00	1.00	0.50	0.50	0.690	0.681	0.430
		**n**	**%**	**n**	**%**	**n**	**%**	CC vs (CT vs TT)	CC vs CT/TT	TT vs CC/CT
	Cigarette smoking, n (%)	14	17.95	6	13.64	2	9.09	0.793	0.813	0.883
	Hypertension, n (%)	12	15.38	6	13.64	0	0.00	0.410	0.656	0.408
	Diabetes mellitus, n (%)	1	1.28	3	6.82	0	0.00	0.195	0.372	0.705
	Gout, n (%)	3	3.85	5	11.36	0	0.00	0.173	0.362	0.884
	Physical therapy, n (%)	33	42.31	19	43.18	4	40.00	0.983	0.974	0.864
	Manual therapy, n (%)	23	29.49	11	25.00	3	30.00	0.860	0.654	0.824
	NSAIDs, n (%)	26	33.33	13	29.55	2	20.00	0.668	0.498	0.667
	Vit. B12 suppl., n (%)	19	24.36	8	18.18	1	10.00	0.483	0.397	0.617

Legend: BMI, body mass index; NSAIDs, nonsteroidal anti-inflammatory drugs; QD, quartile deviation; Vit., vitamin; suppl., supplementation; *, differences remaining significant after Hochberg correction for multiple comparisons (threshold of significance for both additive and recessive/dominant models: $p \le 0.012$).
